# Association between the *PPARGC1A* Gly482Ser (rs8192678) polymorphism and endurance and power athlete status: a systematic review and meta-analysis

**DOI:** 10.3389/fphys.2025.1733458

**Published:** 2026-01-07

**Authors:** Weilong Su, Lingfeng Yuan, Zhaozhe He, Fan Ding, Jun Sun, Yingzhe Xiong, Xiaobo Song

**Affiliations:** 1 School of Physical Education and Sports, Central China Normal University, Wuhan, China; 2 Division of Sports Science and Physical Education, Tsinghua University, Beijing, China; 3 School of Physical Education, Yunnan Normal University, Yunnan, China

**Keywords:** athletic performance, endurance athletes, polymorphism, power athletes, *PPARGC1A*, sport genetics

## Abstract

**Background:**

Evidence on the association between the *PPARGC1A* Gly482Ser (rs8192678) polymorphism and elite athlete status is inconsistent, and a prior meta-analysis has used a genotype-merging approach that may bias results.

**Objective:**

This systematic review and meta-analysis aimed to clarify the association between the *PPARGC1A* Gly482Ser (rs8192678) polymorphism and elite endurance and power athlete status.

**Methods:**

A comprehensive literature search was conducted in PubMed, Web of Science, Embase, and Cochrane Library from inception to November 2025. Studies were included if they provided genotype frequency data for the *PPARGC1A* Gly482Ser polymorphism in elite endurance or power athletes and non-athlete controls. Fixed or random-effects models were used to calculate odds ratios (OR) with 95% confidence intervals (95% CI), and heterogeneity was assessed using the *I* ^*2*^ statistic.

**Results:**

21 studies involving 5,795 athletes and 9,048 non-athlete controls were included. Compared with non-athlete controls, a higher frequency of the Gly/Gly genotype was observed in Caucasian endurance athletes (OR 1.19; 95% CI 1.08–1.31; p < 0.001) and Caucasian power athletes (OR 1.30; 95% CI 1.17–1.44; p < 0.001). In Asians, no significant difference in the frequency of the Gly/Gly genotype was observed between endurance athletes and controls (OR 0.92; 95% CI 0.71–1.19; p = 0.523), whereas a lower frequency was observed in Asian power athletes (OR 0.69; 95% CI 0.53–0.90; p = 0.007).

**Conclusion:**

Our findings demonstrate that the Gly/Gly genotype of the *PPARGC1A* Gly482Ser polymorphism was associated with an increased likelihood of achieving elite athlete status in Caucasians, suggesting its potential as a genetic marker for athletic talent identification in this population. In Asians, no significant association was observed between the *PPARGC1A* Gly482Ser polymorphism and elite endurance athlete status, whereas the Gly/Gly genotype is associated with a lower likelihood of achieving elite power athlete status.

**Systematic Review registration:**

identifier CRD420251148245.

## 1 Introduction

In recent years, there has been a growing body of studies on the influence of genetics on athletic performance, which has significantly contributed to the field of sports science. Approximately 66% of the variation in athletic performance among individuals can be attributed to genetic factors (İpekoğlu et al., 2025). Research indicates that the heritable component of athletic traits may account for up to 90% of variation in anaerobic performance, 60% in cardiorespiratory function, and 70% in maximal muscular strength (Kartibou et al., 2025).

The *PPARGC1A* gene (located on chromosome 4p15.2) encodes the peroxisome proliferator-activated receptor γ coactivator-1a (PGC-1α), a transcriptional coactivator that serves as a key regulator of numerous metabolic pathways (Zhuang et al., 2025). PGC-1α activates transcription factors, such as NRF-1, NRF-2, ERR, PPARγ, RXR, MEF2, FOXO1, HNF-4, and SREBP1, thereby orchestrating multiple mitochondrial and extramitochondrial pathways in cellular energy metabolism. These transcription factors regulate genes involved in mitochondrial biogenesis, fatty acid oxidation, lipogenesis, thermogenesis, and glucose utilization (Michael et al., 2001; Jornayvaz and Shulman, 2010; Huang et al., 2023; Yu et al., 2025). Furthermore, studies have reported that the expression of *PPARGC1A* increases in both rodent and human skeletal muscle following short-term and long-term exercise (Pilegaard et al., 2003; Terada and Tabata, 2004; Mathai et al., 2008; Russell et al., 2003).

In sports science, the most studied polymorphism in the *PPARGC1A* gene is the Gly482Ser (rs8192678) polymorphism, which exerts an influence on both mRNA expression and protein levels (Eynon et al., 2010). The *PPARGC1A* Gly482Ser polymorphism (rs8192678) is a common missense variant that replaces glycine (Gly) with serine (Ser) at codon 482, producing three genotypes: Gly/Gly, Gly/Ser, and Ser/Ser (Hall et al., 2023). Numerous studies have reported associations between this genetic variant and endurance or power athlete status, but findings across different studies are contradictory. For instance, Eynon et al. found the Gly/Gly genotype and Gly allele to be more common in both endurance and power athletes compared with non-athlete controls (Eynon et al., 2011). However, a subsequent study by Grealy et al. analyzing elite Ironman triathletes found no significant association between the *PPARGC1A* Gly482Ser polymorphism and endurance performance (Grealy et al., 2015). Additionally, Gineviciene et al. observed that among Lithuanians, power athletes had a slightly lower frequency of the Gly/Gly genotype and Gly allele than non-athletes (Gineviciene et al., 2016). Although two previous meta-analyses have investigated this association (Chen et al., 2019; Tharabenjasin et al., 2019), genotype-specific evaluations remain limited. Of these, one meta-analysis pooled genotypes that may have opposing effects, an approach that can obscure genotype-specific associations and introduce bias (Tharabenjasin et al., 2019). Additionally, the dataset in Tharabenjasin et al. included athletes from various competitive levels, ranging from college to international athletes. Furthermore, both meta-analyses restricted their literature searches to studies published up to 2018. Together, the emergence of new studies and methodological limitations justify an updated systematic assessment. Therefore, this meta-analysis aims to explore the potential associations between the *PPARGC1A* Gly482Ser polymorphism and elite endurance and power athlete status by meta-analyzing studies on the distribution of genotypes of this polymorphism in endurance and power athletes compared with non-athlete controls.

## 2 Methods

This meta-analysis was registered on PROSPERO with the registration number CRD420251148245 and was reported in accordance with the Preferred Reporting Items for Systematic Reviews and Meta-Analyses (PRISMA) guidelines (Page et al., 2021).

### 2.1 Eligibility criteria

Our analysis includes studies that investigate the association between the *PPARGC1A* Gly482Ser polymorphism and elite endurance or power athlete status, defined as participation at national or international competitive levels. Therefore, studies were included if they met the following criteria: (1) evaluated the association between the *PPARGC1A* Gly482Ser polymorphism and elite endurance or power athlete status; (2) included healthy non-athlete individuals as controls; (3) provided genotype frequency data for the *PPARGC1A* Gly482Ser polymorphism; (4) conformed to the Hardy-Weinberg equilibrium (HWE) in the control group; (5) selected the most recent publication in cases of duplicate data. Studies were excluded for the following reasons: (1) review articles; (2) studies without a control group; (3) absence of genotype frequency data for the *PPARGC1A* Gly482Ser polymorphism; (4) included athletes not competing at national or international level.

### 2.2 Literature search strategy

A comprehensive search was conducted in electronic databases: PubMed, Web of Science, Embase, and Cochrane Library from their inception to November 2025. No restrictions were applied regarding language or publication date. The literature search was conducted using the following key terms: “*PPARGC1A*,” “Peroxisome Proliferator-Activated Receptor Gamma Coactivator 1-alpha,” “PGC-1α,” “rs8192678,” “Gly482Ser,” “polymorphism,” “athletes,” “sports.” Full search terms were provided in Supplementary Table S1. To ensure comprehensive coverage, we also supplemented the electronic search by conducting manual searches of the reference lists of included articles and relevant reviews.

### 2.3 Data extraction

Two authors independently extracted data from all included studies. Any discrepancies were resolved by consulting a third author. Key data were extracted from each of the included studies, including study characteristics (author, publication year, and country), participant characteristics (ethnicity, sample size, sex, age, and athlete status), the number of *PPARGC1A* Gly/Gly, Gly/Ser, and Ser/Ser genotypes in each group, and the genotyping methods.

### 2.4 Quality assessment

The Newcastle–Ottawa Scale (NOS) was used to assess the methodological quality of the studies based on three domains: Selection (four items), Comparability (one item), and Exposure (three items). Each item meeting the criteria was awarded one star, with a maximum possible total of nine stars. Studies could receive up to one star for each item within the Selection and Exposure domains and up to two stars for the Comparability domain. Studies with a score ≥7 were considered high quality. Two authors independently conducted the quality assessment, and any disagreements were resolved through consultation with a third author.

### 2.5 Statistical analyses

The association between the *PPARGC1A* Gly482Ser polymorphism and elite athlete status was determined by calculating pooled odds ratios (OR) with 95% confidence intervals (95% CI). Statistical significance was defined as a *p*-value of ≤0.05. The statistical heterogeneity across the included studies was assessed using the *I* ^*2*^ statistic. *I* ^*2*^ values of 25%, 50%, and 75% represented low, moderate, and high heterogeneity respectively. The heterogeneity level determined model selection: fixed-effects models were applied when *I* ^*2*^ < 50%, while random-effects models were used when *I* ^*2*^ > 50%. Publication bias in our meta-analysis was assessed through visual inspection of funnel plots for all comparisons. Sensitivity analyses were performed by sequentially removing each study to assess the stability of the overall results. This meta-analysis was conducted using Stata 18.0.

## 3 Results

### 3.1 Selection of studies

**FIGURE 1:**
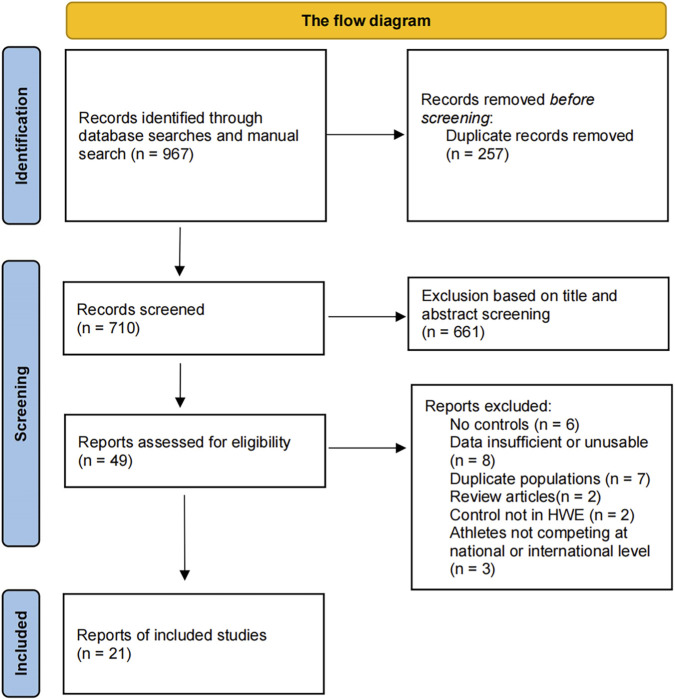
The flow diagram of included/excluded studies.

A total of 967 records were identified through database searches and manual search. After removing duplicates (n = 257) and excluding studies based on the title and abstract screening (n = 661), 49 studies remained. Based on the full-text assessment, a further 29 studies were excluded for the following reasons: no controls (n = 6), data insufficient or unusable (n = 8), duplicate populations (n = 7), review articles (n = 2), control not in HWE (n = 2), and athletes not competing at national or international level (n = 3). Finally, 21 eligible studies were included in the systematic review and meta-analysis (Figure 1).

### 3.2 Characteristics of the included studies

These studies involved a total of 5,795 athletes (comprising 3,351 endurance athletes and 2,444 power athletes) and 9,048 controls. According to the type of sports, athletes were divided into endurance and power groups. The endurance group included marathon, biathlon, orienteering, steeplechase, long-distance swimming, football, pentathlon, rowing, road cycling, cross-country skiing, long-distance track and field athletics, triathlon, long-distance speed skating, race walking, and mountain biking. The power group included weightlifting, short-distance track and field athletics, powerlifting, kayaking, judo, wrestling, boxing, fencing, short-distance swimming, speed skating, alpine skiing, artistic gymnastics, throwing events, jumping events, bodybuilding, ski jumping, canoe speed, basketball, volleyball, tennis, hockey and decathlon. The characteristics of the included studies are summarized in Table 1. Distributions of the *PPARGC1A* Gly482Ser polymorphism genotypes in endurance and power athletes across included studies are presented in Supplementary Table S2; Supplementary Table S3 respectively.

**TABLE 1:** Characteristics of included studies in the systematic review and meta-analysis.

Study	Country	Race	Athlete status	Total athletes	Total controls	NOS
Bulğay et al. (2022)	Turkey	C	National endurance and power athletes	Number: 60 Sex: Data not shown Age: 25.07 ± 4.80	Number: 20 Sex: Data not shown Age: 23.51 ± 7.13	7
Hall et al. (2023)	Britain	C	National endurance athletes	Number: 288 Sex: 201 men, 87 women Age: Data not shown	Number: 368 Sex: 285 men, 83 women Age: Data not shown	7
Eynon et al. (2011)	Israel	C	Olympic-class or national endurance and power athletes	Number: 155 Sex: 119 men, 36 women Age: 35.9 ± 12.2	Number: 240 Sex: 170 men,70 women Age: 26 ± 3	8
Ginevičienė et al. (2011)	Lithuania	C	International, national or regional endurance and power athletes	Number: 128 Sex: 106 men, 22 women Age: 22 ± 6.3	Number: 250 Sex: 167 men, 83 women Age: 36.2 ± 7.2	9
Gineviciene et al. (2014)	Lithuania	C	International or national footballers	Number: 199 Sex: 199 men Age: 17–20	Number: 167 Sex: 167 men Age: 18–22	8
Gineviciene et al. (2016)	Russia	C	International or national power athletes	Number: Russian: 114 Lithuanian: 47 Sex: 128 men, 33 women Age: 23 ± 6.5	Number: Russian: 947 Lithuanian: 255 Sex: 540 men, 662 women Age: 29 ± 8.5	8
	Lithuania	C				
Grealy et al. (2015)	Australia	C	International endurance triathletes	Number: 195 Sex: 143 men, 52 women Age: 42.5 ± 11.4	Number: 113 Sex: Data not shown Age: Data not shown	6
Guilherme and Lancha (2020)	Brazil	C	International or national power athletes	Number: 83 Sex: Data not shown Age: Data not shown	Number: 818 Sex: 472 men, 346 women Age: 32.6 ± 12.3	7
Guilherme et al. (2018)	Brazil	C	Endurance and power athletes of the Brazilian national team	Number: 630 Sex: 426 men, 204 women Age: 27.6 ± 10.5	Number: 893 Sex: 521 men, 372 women Age: 35.1 ± 17.6	7
He et al. (2015)	China	A	International or national endurance athletes	Number: 235 Sex: 108 men, 127 women Age: 23 ± 4 (men) 21 ± 4 (women)	Number: 504 Sex: 267 men, 237 women Age: 20 ± 1	7
Homma et al. (2022)	Japan	A	International or national weightlifters	Number: 192 Sex: 113 men, 79 women Age: 22.45 ± 8.25	Number: 416 Sex: 151 men, 265 women Age: 52.27 ± 18.02	8
			International or national power athletes	Number: 177 Sex: 144 men, 33 women Age: Data not shown	Number: 416 Sex: 151 men, 265 women Age: 52.27 ± 18.02	
Lucia et al. (2005)	Spain	C	Olympic-class riders, middle-distance and long-distance track runners	Number: 104 Sex: 104 men Age: Data not shown	Number: 100 Sex: 100 men Age: 18–55	7
Maciejewska et al. (2012)	Poland	C	Olympic-class endurance and power athletes	Number: 302 Sex: 221 men, 81 women Age: 27.8 ± 7.1	Number: 684 Sex: Data not shown Age: 19–23	8
	Russia	C		Number:1,303 Sex:888 men, 415 women Age: 24.4 ± 0.3	Number:1,132 Sex:537 men, 595 women Age: 17.2 ± 0.2	
Maruszak et al. (2014)	Poland	C	Olympic-class or national endurance and power athletes	Number: 395 Sex: 249 men, 146 women Age: Data not shown	Number: 413 Sex: Data not shown Age: Data not shown	7
Muniesa et al. (2010)	Spain	C	Olympic-class runners, riders and rowers	Number: 141 Sex: 141 men Age: Data not shown	Number: 123 Sex: 123men Age: Data not shown	8
Peplonska et al. (2017)	Poland	C	Olympic-class or national endurance and power athletes	Number: 413 Sex: 263 men, 150 women Age: 23.5 ± 4.7	Number: 451 Sex: 217 men, 234 women Age: 23 ± 3.1	7
Santiago et al. (2010)	Spain	C	National rowers	Number: 15 Sex: 15 men Age: Data not shown	Number: 123 Sex: 123men Age: Data not shown	8
Varillas Delgado et al. (2020)	Spain	C	International road cyclists and runners	Number: 123 Sex: 123 men Age: 24.9 ± 4.9	Number: 122 Sex: 122 men Age: 27.9 ± 4.5	8
Varillas-Delgado et al. (2022)	Spain	C	International or national endurance athletes	Number: 292 Sex: 292 men Age: 25.8 ± 4.2	Number: 160 Sex: 160 men Age: 25.8 ± 4.2	8
Yvert et al. (2016)	Japan	A	Olympic-class or national endurance athletes	Number: 154 Sex: 97 men, 57 women Age: Data not shown	Number: 649 Sex: 184 men, 465 women Age: Data not shown	8
Valipour et al. (2021)	Iran	C	Athletes of the Iranian national hockey team	Number: 50 Sex: Data not shown Age: Data not shown	Number: 100 Sex: Data not shown Age: Data not shown	6

NOS, Newcastle–Ottawa Scale; C, caucasian; A, asian.

### 3.3 Study quality assessment

Following the assessment using the Newcastle–Ottawa Scale (NOS), 2 study scored 6 points, 8 studies scored 7 points, 10 studies scored 8 points, and 1 study scored 9 points (Table 1). With a mean score of 7.48 ± 0.74, the overall quality of the evidence was considered satisfactory.

### 3.4 Meta-analysis

#### 3.4.1 Endurance athletes

The distribution of *PPARGC1A* Gly/Gly, Gly/Ser, and Ser/Ser genotypes in endurance athletes is as follows. A higher frequency of the Gly/Gly genotype was observed compared with the Gly/Ser genotype in Caucasians (OR 1.35; 95% CI 1.06–1.71; *p* = 0.015; 78.7% heterogeneity) (Supplementary Figure S1), whereas a lower frequency of the Gly/Gly genotype was observed in Asians (OR 0.41; 95% CI 0.30–0.57; *p* < 0.001; 9% heterogeneity) (Supplementary Figure S1). When combining Caucasian and Asian populations, no significant difference between the Gly/Gly genotype and the Gly/Ser genotype was detected (OR 1.16; 95% CI 0.88–1.53; *p* = 0.301; 86.6% heterogeneity) (Supplementary Figure S1). Compared with the Ser/Ser genotype, the Gly/Ser genotype was significantly more frequent in the combined populations (OR 7.21; 95% CI 5.30–9.81; *p* < 0.001; 74.8% heterogeneity) (Supplementary Figure S2). This advantage was more pronounced in Caucasians (OR 8.14; 95% CI 5.93–11.16; *p* < 0.001; 69.3% heterogeneity) (Supplementary Figure S2) than in Asians (OR 3.49; 95% CI 2.55–4.76; *p* < 0.001; 0% heterogeneity) (Supplementary Figure S2). Similarly, the Gly/Gly genotype showed a significantly higher frequency than the Ser/Ser genotype in the combined populations (OR 8.79; 95% CI 5.25–14.74; *p* < 0.001; 91.4% heterogeneity) (Supplementary Figure S3), with Caucasians showing the same trend (OR 11.05; 95% CI 7.16–17.06; *p* < 0.001; 84.2% heterogeneity) (Supplementary Figure S3), whereas no statistically significant association was observed in Asians (OR 1.39; 95% CI 0.81–2.40; *p* = 0.229; 62.4% heterogeneity) (Supplementary Figure S3).

**FIGURE 2:**
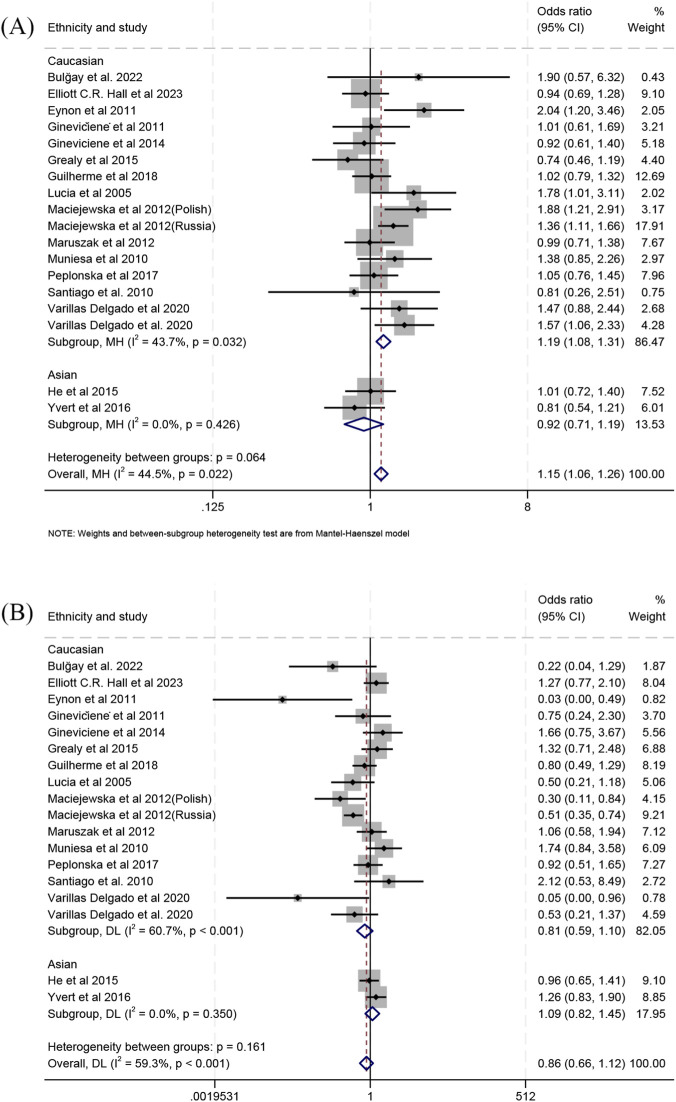
Forest plot of the comparison between genotype frequencies in endurance athletes versus controls. **(A)** Gly/Gly vs. Gly/Ser + Ser/Ser and **(B)** Ser/Ser vs. Gly/Gly + Gly/Ser.

When comparing endurance athletes with controls, we found a higher frequency of the Gly/Gly genotype in Caucasian athletes (OR 1.19; 95% CI 1.08–1.31; *p* < 0.001; 43.7% heterogeneity) (Figure 2A), whereas no significant association was detected in Asians (OR 0.92; 95% CI 0.71–1.19; *p* = 0.523; 0% heterogeneity) (Figure 2A).When combining both populations, the Gly/Gly genotype frequency was higher in athletes than controls (OR 1.15; 95% CI 1.06–1.26; *p* = 0.001; 44.5% heterogeneity) (Figure 2A). Analyses of the Ser/Ser genotype showed no statistically significant differences: Caucasians (OR 0.81; 95% CI 0.59–1.10; *p* = 0.179; 60.7% heterogeneity) (Figure 2B), Asians (OR 1.09; 95% CI 0.82–1.45; *p* = 0.548; 0% heterogeneity) (Figure 2B), or the combined populations (OR 0.86; 95% CI 0.66–1.12; *p* = 0.263; 59.3% heterogeneity) (Figure 2B). In allelic comparisons, a higher frequency of the Gly allele was detected in the combined populations (OR 1.15; 95% CI 1.01–1.30; *p* = 0.032; 67% heterogeneity) (Supplementary Figure S4) and Caucasians (OR 1.19; 95% CI 1.04–1.36; *p* = 0.014; 66.5% heterogeneity) (Supplementary Figure S4). However, no significant differences were found in Asians (OR 0.94; 95% CI 0.79–1.12; *p* = 0.468; 9.6% heterogeneity) (Supplementary Figure S4).

**FIGURE 3:**
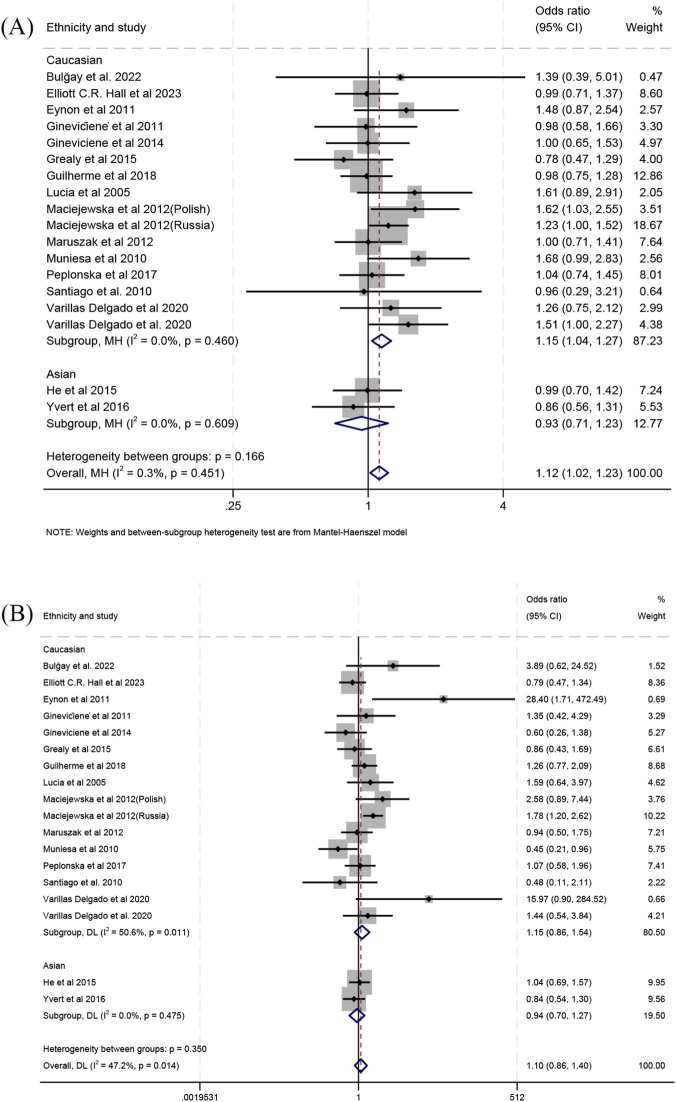
Forest plot of the comparison between individual genotype frequencies in endurance athletes versus controls. **(A)** Gly/Gly vs. Gly/Ser and **(B)** Gly/Ser vs. Ser/Ser.

When comparing individual genotypes between endurance athletes and controls, the frequency of carrying the Gly/Gly genotype relative to the Gly/Ser genotype was higher in endurance athletes than in controls (OR 1.12; 95% CI 1.02–1.23; *p* = 0.016; 0.3% heterogeneity) (Figure 3A), with Caucasians showing the same trend (OR 1.15; 95% CI 1.04–1.27; *p* = 0.006; 0% heterogeneity) (Figure 3A) and Asians exhibiting no significant difference (OR 0.93; 95% CI 0.71–1.23; *p* = 0.628; 0% heterogeneity) (Figure 3A). In the comparison of Gly/Ser and Ser/Ser genotypes, no significant difference was observed between endurance athletes and controls in Caucasians (OR 1.15; 95% CI 0.86–1.54; *p* = 0.351; 50.6% heterogeneity) (Figure 3B), Asians (OR 0.94; 95% CI 0.70–1.27; *p* = 0.694; 0% heterogeneity) (Figure 3B), or the combined populations (OR 1.10; 95% CI 0.86–1.40; *p* = 0.449; 47.2% heterogeneity) (Figure 3B). Similarly, In the comparison of Gly/Gly and Ser/Ser genotypes, no significant difference was found for Caucasians (OR 1.35; 95% CI 0.95–1.91; *p* = 0.090; 64.4% heterogeneity) (Supplementary Figure S5), Asians (OR 0.88; 95% CI 0.62–1.25; *p* = 0.479; 12.4% heterogeneity) (Supplementary Figure S5), or the combined populations (OR 1.24; 95% CI 0.92–1.67; *p* = 0.154; 64% heterogeneity) (Supplementary Figure S5).

#### 3.4.2 Power athletes

The distribution of *PPARGC1A* Gly/Gly, Gly/Ser, and Ser/Ser genotypes in power athletes is as follows. Compared with the Gly/Ser genotype, the Gly/Gly genotype was more frequent in Caucasians (OR 1.47; 95% CI 1.11–1.93; *p* = 0.006; 72.5% heterogeneity) (Supplementary Figure S6), but less frequent in Asians (OR 0.36; 95% CI 0.26–0.48; *p* < 0.001; 0% heterogeneity) (Supplementary Figure S6) and showed no significant association in the combined populations (OR 1.13; 95% CI 0.78–1.64; *p* = 0.516; 88.6% heterogeneity) (Supplementary Figure S6). The frequency of the Gly/Ser genotype was significantly higher than the Ser/Ser genotype in Caucasians (OR 6.80; 95% CI 5.70–8.11; *p* < 0.001; 37.2% heterogeneity) (Supplementary Figure S7), Asians (OR 5.11; 95% CI 3.66–7.14; *p* < 0.001; 0% heterogeneity) (Supplementary Figure S7), and the combined populations (OR 6.42; 95% CI 5.50–7.50; *p* < 0.001; 32.3% heterogeneity) (Supplementary Figure S7). Similarly, the frequency of the Gly/Gly genotype was higher than the Ser/Ser genotype in Caucasians (OR 10.41; 95% CI 7.57–14.32; *p* < 0.001; 57.9% heterogeneity) (Supplementary Figure S8), Asians (OR 1.82; 95% CI 1.28–2.57; *p =* 0.001; 0% heterogeneity) (Supplementary Figure S8), and the combined populations (OR 8.13; 95% CI 5.06–13.05; *p* < 0.001; 86.4% heterogeneity) (Supplementary Figure S8).

**FIGURE 4:**
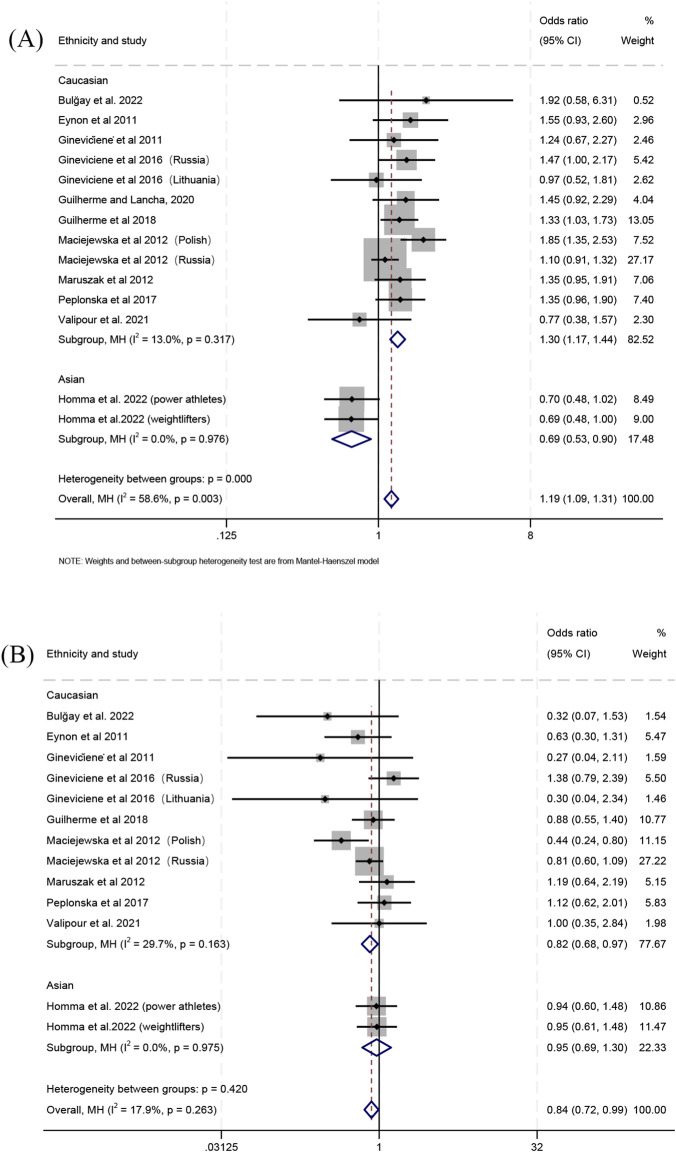
Forest plot of the comparison between genotype frequencies in power athletes versus controls. **(A)** Gly/Gly vs. Gly/Ser + Ser/Ser and **(B)** Ser/Ser vs. Gly/Gly + Gly/Ser.

When comparing power athletes with controls, the Gly/Gly genotype was more frequent in power athletes in the combined populations (OR 1.19; 95% CI 1.09–1.31; *p* < 0.001; 58.6% heterogeneity) (Figure 4A) and in Caucasians (OR 1.30; 95% CI 1.17–1.44; *p* < 0.001; 13% heterogeneity) (Figure 4A), but less frequent in Asians (OR 0.69; 95% CI 0.53–0.90; *p* = 0.007; 0% heterogeneity) (Figure 4A). In contrast, a lower frequency of the Ser/Ser genotype was observed in the combined populations (OR 0.84; 95% CI 0.72–0.99; *p* = 0.033; 17.9% heterogeneity) and in Caucasians (OR 0.82; 95% CI 0.68–0.97; *p* = 0.025; 29.7% heterogeneity) (Figure 4B), whereas no significant difference was detected in Asians (OR 0.95; 95% CI 0.69–1.30; *p* = 0.733; 0% heterogeneity) (Figure 4B). Furthermore, there was a higher frequency of the Gly allele in the combined populations (OR 1.15; 95% CI 1.07–1.24; *p* < 0.001; 49.4% heterogeneity) (Supplementary Figure S9) and in Caucasians (OR 1.22; 95% CI 1.13–1.32; *p* < 0.001; 18.2% heterogeneity) (Supplementary Figure S9), but no significant difference was detected in Asians (OR 0.86; 95% CI 0.72–1.03; *p* = 0.099; 0% heterogeneity) (Supplementary Figure S9).

**FIGURE 5:**
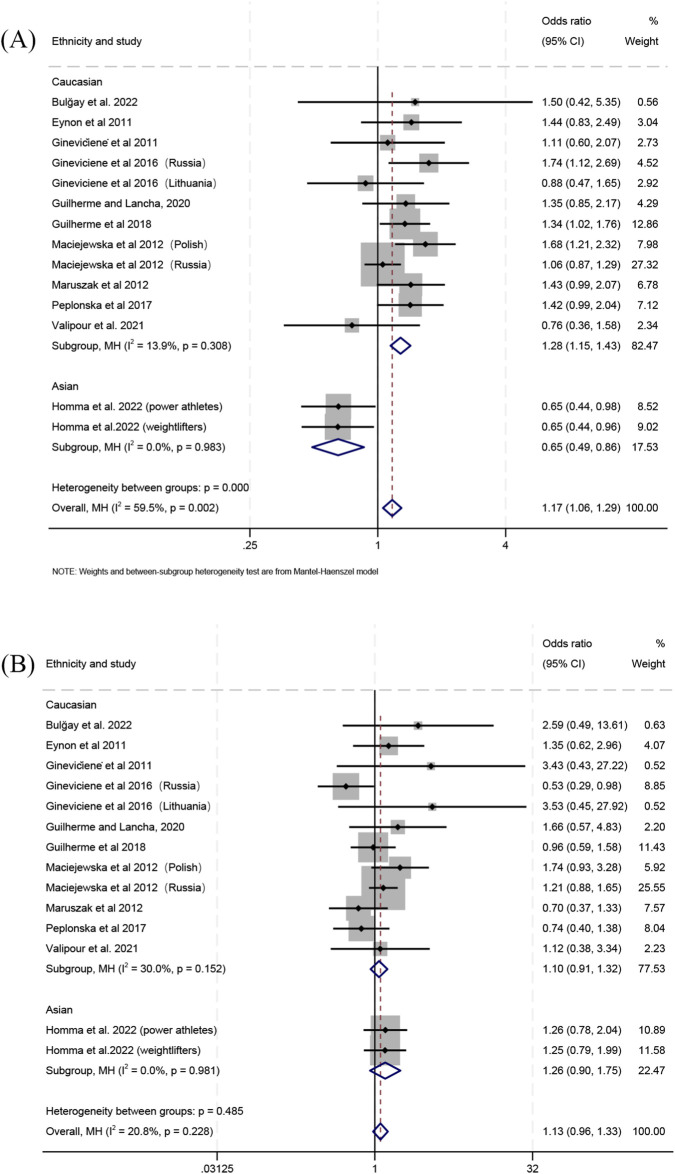
Forest plot of the comparison between individual genotype frequencies in power athletes versus controls. **(A)** Gly/Gly vs. Gly/Ser and **(B)** Gly/Ser vs. Ser/Ser.

When comparing individual genotypes between power athletes and controls, the frequency of the Gly/Gly genotype relative to the Gly/Ser genotype was higher in the combined populations (OR 1.17; 95% CI 1.06–1.29; *p* = 0.002; 59.5% heterogeneity) (Figure 5A) and in Caucasians (OR 1.28; 95% CI 1.15–1.43; *p* < 0.001; 13.9% heterogeneity) (Figure 5A), but lower in Asians (OR 0.65; 95% CI 0.49–0.86; *p* = 0.003; 0% heterogeneity) (Figure 5A). For the comparison of the Gly/Ser and Ser/Ser genotypes, no significant differences between power athletes and controls were detected in Caucasians (OR 1.10; 95% CI 0.91–1.32; *p* = 0.332; 30% heterogeneity) (Figure 5B), Asians (OR 1.26; 95% CI 0.90–1.75; *p* = 0.181; 0% heterogeneity) (Figure 5B) or the combined populations (OR 1.13; 95% CI 0.96–1.33; *p* = 0.134; 20.8% heterogeneity) (Figure 5B). Additionally, the frequency of the Gly/Gly genotype relative to the Ser/Ser genotype was higher in power athletes than in controls for the combined populations (OR 1.26; 95% CI 1.07–1.48; *p* = 0.005; 37.7% heterogeneity) (Supplementary Figure S10) and Caucasians (OR 1.40; 95% CI 1.17–1.68; *p* < 0.001; 25.8% heterogeneity) (Supplementary Figure S10), whereas no significant difference was observed in Asians (OR 0.82; 95% CI 0.57–1.17; *p* = 0.273; 0% heterogeneity) (Supplementary Figure S10).

### 3.5 Sensitivity analysis and publication bias

To assess the effect of each study on the overall results, we conducted sensitivity analysis. The use of fixed-effects and random-effects models was based on the percentage of heterogeneity. After excluding one study each time, the ORs, 95% confidence interval, and p-values did not significantly change, which may indicate the robustness of our results. Moreover, the visual inspection of the funnel plots suggested no publication bias among the included studies. The information of funnel plots is provided in Supplementary Figures S11–S29.

## 4 Discussion

The primary objective of this meta-analysis was to investigate the associations between the *PPARGC1A* Gly482Ser polymorphism and athlete status, focusing on genotype distribution in endurance and power athletes compared with controls. In Caucasians, the distribution of the *PPARGC1A* Gly482Ser polymorphism in both endurance and power athletes followed the trend: Gly/Gly > Gly/Ser > Ser/Ser. Furthermore, both the Gly/Gly genotype and the Gly allele were significantly more frequent in Caucasian elite athletes compared with controls. Specifically, the Gly/Gly genotype was more frequently observed than the Gly/Ser genotype in both endurance and power athletes compared with controls. In contrast, direct comparisons of Gly/Ser versus Ser/Ser revealed no significant differences between athletes and controls in either discipline.

Notably, three studies respectively investigating Asian athletes from China and Japan (He et al., 2015; Yvert et al., 2016; Homma et al., 2022), included 758 athletes (389 endurance athletes and 369 power athletes). For endurance athletes, there is no statistically significant differences in genotype frequencies (Gly/Gly, Gly/Ser, Ser/Ser) compared with their respective controls. For power athletes, the Gly/Gly genotype is less frequent relative to controls, whereas the Gly/Ser and Ser/Ser genotypes show no significant differences between athletes and controls. These findings suggest that the *PPARGC1A* Gly482Ser polymorphism may not be associated with Asian athlete status. However, this conclusion should be interpreted with caution. The Genome Aggregation Database (gnomAD) reveals significant inter-population differences in rs8192678 allele frequencies (Chen et al., 2024). The Ser allele is more common in East Asians, less frequent in Caucasians, and rare in African populations. Consequently, the baseline prevalence of the Gly/Gly genotype is lower in East Asians than in Caucasians. These background differences, combined with the limited sample size of existing Asian athlete cohorts, may reduce statistical power to detect genotype–phenotype effects, which may partly explain the absence of significant associations in our meta-analysis.

Our findings demonstrate that the Gly/Gly genotype is advantageous for both endurance and power athletic performance. This finding is consistent with two previous meta-analyses (Chen et al., 2019; Tharabenjasin et al., 2019) and is further supported, for endurance-specific cohorts, by a homogeneous meta-analysis of long-distance runners and road cyclists (Konopka et al., 2022).Mechanistically, this genotype enhances the expression of PGC-1α, a key transcriptional coactivator that regulates cellular energy metabolism. For endurance athletes, enhanced PGC-1α expression promotes GLUT4-mediated glucose transport and muscle glycogen storage, ultimately improving metabolic efficiency and athletic performance (Katz et al., 1986; Wasserman, 2009; Wasserman et al., 2011; Richter and Hargreaves, 2013). Furthermore, PGC-1α associates with and coactivates nuclear respiratory factor 1 (NRF-1), thereby upregulating the expression of mitochondrial transcription factor A (mtTFA), which directly activates the transcription and replication of mitochondrial DNA. One study found that cells overexpressing PGC-1α exhibited a 57% increase in mitochondrial density relative to control cells (Wu et al., 1999). This enhanced skeletal muscle mitochondrial content can lead to reduced lactate production, enhanced fat utilization, and improved endurance performance (Coyle et al., 1988; LeBlanc et al., 2004). Furthermore, this increased oxidative capacity also elevates the rate of carbohydrate oxidation when required, enabling higher power output and contributing to improved athletic performance (Westgarth-Taylor et al., 1997). Upregulation of PGC-1α has been shown to significantly enhance fatty acid oxidation in skeletal muscle, which reduces reliance on finite muscle glycogen reserves, prolongs exercise duration, and ultimately improves endurance performance (Huang et al., 2017; Muscella et al., 2020; Yu et al., 2025; Gerhart-Hines et al., 2007). Moreover, PGC-1α expression induces a functional shift in skeletal muscle, converting fast-twitch type IIb fibers toward a more oxidative type IIa and type I fibers (Lin et al., 2002).

Notably, the same metabolic adaptations also contribute to enhanced power performance. The increase in glucose transport and muscle glycogen storage provides sufficient substrate for the glycolytic system, thereby supporting high-power output. Moreover, the elevated mitochondrial content reduces lactate accumulation during high-intensity efforts (Chesley et al., 1996). Additionally, the improved oxidative capacity is a key factor determining the rate of PCr resynthesis and the restoration of performance during repeated sprint exercises (Bogdanis et al., 1996). Studies have demonstrated the aerobic system contributes substantially to energy production even in high-intensity efforts: Spencer and Gastin et al. revealed that the aerobic system is activated rapidly after the onset of exercise and becomes the predominant energy supplier between 15 and 30 s for the 400-m, 800-m, and 1500-m events (Spencer and Gastin, 2001). Similarly, Hargreaves and Spriet et al. demonstrated that aerobic ATP production is activated during very intense exercise, with approximately 50% of the energy contribution in the final 5 s of a 30-s sprint being derived aerobically (Hargreaves and Spriet, 2020). Moreover, achieving elite athlete status depends not only on athletic performance capacity but also on the risk of sports-related injuries and the ability to recover from them. Evidence from candidate-gene and genome-wide association studies indicates that genetic background contributes to inter-individual variability in susceptibility to musculoskeletal injuries and in the rate of recovery (Koks et al., 2020; Ebert et al., 2023; Massidda et al., 2024; Sun et al., 2025). Several experimental studies using acute skeletal muscle injury models have shown that muscle-specific overexpression of PGC-1α leads to smaller necrotic areas, faster clearance of necrotic debris, and better preservation of muscle architecture after injury (Dinulovic et al., 2016; Washington et al., 2022). Given that power athletes are at a particularly high risk of acute musculoskeletal injuries, we hypothesized that the Gly/Gly genotype would confer an additional advantage in this population by mitigating the risk of such injuries and/or facilitating faster recovery, thereby contributing to their likelihood of achieving elite status.

Our findings also demonstrate that individuals carrying one or two copies of the Ser allele of the *PPARGC1A* Gly482Ser polymorphism show no significant association with elite endurance athlete status and exhibit a lower likelihood of achieving elite power athlete status. This finding diverges from the conclusions of two prior meta-analyses (Chen et al., 2019; Tharabenjasin et al., 2019). Potential explanations for this discrepancy include the larger sample size incorporated in the present meta-analysis and potential differences in population genetic backgrounds. In addition, the previously reported association may have been confounded by the strong selective advantage of the Gly/Gly genotype, which increases the overall Gly allele frequency in athletes rather than reflecting a true effect of the heterozygous genotype. To date, the functional impact of the Ser allele remains controversial. Several studies support an impaired functional role associated with the Ser allele. For instance, Stefan et al. found that the Gly/Ser or Ser/Ser genotype in *PPARGC1A* was associated with lower aerobic fitness (Stefan et al., 2007), and Petr et al. reported that carriers of the Ser allele exhibited reduced training responsiveness after aerobic training (Petr et al., 2018). Moreover, Steinbacher et al. reported that the Ser-encoding allele inhibits the exercise-induced transition from type II to type I muscle fibers, although it does not affect improvements in mitochondrial biogenesis, capillarization, or lipid metabolism (Steinbacher et al., 2015). In contrast, other studies report no functional deficit under certain conditions. Okauchi et al. demonstrated that no significant difference in the level of co-activation was observed between the wild-type (Gly482) and variant (Ser482) PGC-1α proteins (Okauchi et al., 2008). Furthermore, one study revealed that the Ser482 allele exerted no significant influence on PGC-1α mRNA expression levels in young individuals but was associated with reduced expression in elderly carriers (Ling et al., 2004), suggesting an age-dependent functional impact of the Ser allele.

This study identifies methodological limitations in the approach of Tharabenjasin et al. who merged antagonistic genotypes (Gly/Gly + Ser/Ser) into a single reference group, which may introduce bias (Tharabenjasin et al., 2019). First, because genotype frequencies sum to 100%, the beneficial effect of the Gly/Gly genotype will necessarily reduce the proportions of the remaining genotypes in athletes. Thus, a lower frequency of Gly/Ser in athletes relative to controls cannot be interpreted as evidence that it is disadvantageous; it may instead reflect the dominance of Gly/Gly genotype. Second, combining opposite-effect genotypes (Gly/Gly + Ser/Ser) distorts the baseline of the reference group. For instance, if the Gly/Ser genotype has an enhancing or neutral effect on athletic performance, the Gly/Gly genotype is significantly more frequent in athletes than in controls, and the Ser/Ser genotype is slightly less frequent. Consequently, the combined frequency of Gly/Gly + Ser/Ser genotypes may be higher in athletes than in controls. This results in the frequency of the Gly/Ser genotype in athletes appearing relatively lower than in controls. Alternatively, if the Gly/Ser genotype has a detrimental effect on athletic performance and the frequency of Gly/Gly + Ser/Ser genotypes is lower in athletes than in controls. This results in the frequency of the Gly/Ser genotype in athletes appearing relatively higher than in controls. Therefore, to avoid confounding effects arising from the opposing influences of Gly/Gly and Ser/Ser genotypes, the present study separately compared Gly/Gly versus Gly/Ser, Gly/Ser versus Ser/Ser, and Gly/Gly versus Ser/Ser to reevaluate the impact of the Gly/Ser genotype on athletic performance. Furthermore, as noted by Chen et al., the study by Tharabenjasin et al. incorporated duplicate genotype data from overlapping populations (Chen et al., 2019), further underscoring the need for a re-evaluation of this topic.

This meta-analysis has several limitations. First, due to the scarcity of studies on the *PPARGC1A* Gly482Ser polymorphism in Asian populations, particularly among power athletes, our findings are primarily applicable to Caucasians. Second, although we dichotomized athletic disciplines into “power” and “endurance” groups for this meta-analysis, this approach may oversimplify the complex physiological demands of different sports. Additionally, we observed high heterogeneity in some of the analyzed groups. Despite conducting subgroup analyses by ethnicity and genotyping method, the sources of heterogeneity could not be definitively identified. Finally, due to the unavailability of comprehensive individual data from the included studies, we were unable to perform sex-based subgroup analyses. This represents a critical shortcoming, considering that sex exerts a profound influence on both gene expression and athletic performance phenotypes (Singh et al., 2018; Senefeld and Hunter, 2024; Hunter et al., 2023). For instance, Mägi et al. reported that the ACE ID and ACTN3 RR genotypes were significantly associated with elite male skier status in a longitudinal cohort, but no such association was observed in females (Mägi et al., 2016).

Our findings suggest that the Gly/Gly genotype of the *PPARGC1A* Gly482Ser polymorphism may represent a candidate genetic marker associated with elite athlete status in Caucasians. Therefore, screening for the Gly/Gly genotype in youth talent identification and elite athlete development programs could contribute to a more scientific evaluation of genetic predisposition and optimize the allocation of training resources. These findings provide strong evidence for the role of genetics in athletic performance. Meanwhile, the lack of a significant association in Asian endurance athletes highlights the need for population-specific approaches in genetic profiling. To improve the accuracy of talent identification, we recommend integrating this marker with other sports-related genes into polygenic scores.

In conclusion, the association between the *PPARGC1A* Gly482Ser polymorphism and elite athlete status appears population-specific. In Caucasians, the Gly/Gly genotype of the *PPARGC1A* Gly482Ser polymorphism was associated with an increased likelihood of achieving elite athlete status, whereas the Gly/Ser and Ser/Ser genotypes showed no significant association with elite endurance athlete status and were associated with a lower likelihood of achieving elite power athlete status. In Asians, no significant association was observed between the *PPARGC1A* Gly482Ser polymorphism and elite endurance athlete status. The Gly/Gly genotype is associated with a lower likelihood of achieving elite power athlete status, whereas the Gly/Ser and Ser/Ser genotypes show no significant association with elite power athlete status. In this meta-analysis, we resolve methodological limitations in a previous meta-analysis (Tharabenjasin et al., 2019), thereby providing a more accurate evaluation. Future studies should enroll diverse ethnic cohorts, report data stratified by sex and conduct functional experiments to elucidate how the *PPARGC1A* polymorphisms modulate energy metabolism and athletic performance.

## Data Availability

The datasets presented in this study can be found in online repositories. The names of the repository/repositories and accession number(s) can be found in the article/Supplementary Material.
