# Supplementary material for: Association between the PPARGC1A Gly482Ser (rs8192678) polymorphism and endurance and power athlete status: a systematic review and meta-analysis
Source: Front Physiol. 2026 Jan 7;16:1733458. doi: 10.3389/fphys.2025.1733458 (PMC12819181; doi:10.3389/fphys.2025.1733458)
Supplement: Supplementary file 1 [file DataSheet1.docx]

**Supplementary Table 1.** Search terms.

| **Database** | **Search terms** |
| --- | --- |
| PubMed | (("Peroxisome Proliferator-Activated Receptor Gamma Coactivator 1-alpha"[Mesh]) OR (Peroxisome Proliferator Activated Receptor Gamma Coactivator 1 alpha[Title/Abstract]) OR (PPARGC-1-alpha Protein[Title/Abstract]) OR (PPARGC 1 alpha Protein[Title/Abstract]) OR (PGC-1-alpha Protein[Title/Abstract]) OR (PGC 1 alpha Protein[Title/Abstract]) OR (PPARGC1a Protein[Title/Abstract]) OR (rs8192678[Title/Abstract])) AND (("Sports"[Mesh]) OR (Sport[Title/Abstract]) OR (Athletics[Title/Abstract]) OR (Athletic[Title/Abstract]) OR ("Athletes"[Mesh]) OR (Athlete[Title/Abstract]) OR (Professional Athletes[Title/Abstract]) OR (Athlete, Professional[Title/Abstract]) OR (Athletes, Professional[Title/Abstract]) OR (Professional Athlete[Title/Abstract]) OR (Elite Athletes[Title/Abstract]) OR (Athlete, Elite[Title/Abstract]) OR (Athletes, Elite[Title/Abstract]) OR (Elite Athlete[Title/Abstract]) OR (College Athletes[Title/Abstract]) OR (Athlete, College[Title/Abstract]) OR (Athletes, College[Title/Abstract]) OR (College Athlete[Title/Abstract]) OR (endurance elite athletes[Title/Abstract]) OR (endurance sub-elite athletes[Title/Abstract]) OR (endurance athletes[Title/Abstract]) OR (endurance individual sports athletes[Title/Abstract]) OR (endurance team sports athletes[Title/Abstract]) OR (power elite athletes[Title/Abstract]) OR (power sub-elite athletes[Title/Abstract]) OR (power athletes[Title/Abstract]) OR (power individual sports athletes[Title/Abstract]) OR (power team sports athletes[Title/Abstract])) |
| Web of Science | ((((((((TS=(Peroxisome Proliferator-Activated Receptor Gamma Coactivator 1-alpha)) OR AB=(Peroxisome Proliferator Activated Receptor Gamma Coactivator 1 alpha)) OR AB=(PPARGC-1-alpha Protein)) OR AB=(PPARGC 1 alpha Protein)) OR AB=(PGC-1-alpha Protein)) OR AB=(PGC 1 alpha Protein)) OR AB=(PPARGC1a Protein))) AND ((((((((((((((((((((((((((((TS=(Sports)) OR AB=(Sport)) OR AB=(Athletics)) OR AB=(Athletic)) OR TS=(Athletes) OR AB=(Athlete)) OR AB=(Professional Athletes)) OR AB=(Athlete, Professional)) OR AB=(Athletes, Professional)) OR AB=(Professional Athlete)) OR AB=(Elite Athletes)) OR AB=(Athlete, Elite)) OR AB=(Athletes, Elite)) OR AB=(Elite Athlete)) OR AB=(College Athletes)) OR AB=(Athlete, College)) OR AB=(Athletes, College)) OR AB=(College Athlete)) OR AB=(endurance elite athletes)) OR AB=(endurance sub-elite athletes)) OR AB=(endurance athletes)) OR AB=(endurance individual sports athletes)) OR AB=(endurance team sports athletes)) OR AB=(power elite athletes)) OR AB=(power sub-elite athletes)) OR AB=(power athletes)) OR AB=(power individual sports athletes)) OR AB=(power team sports athletes))) |
| Embase | ('peroxisome proliferator activated receptor gamma coactivator 1alpha'/exp OR 'peroxisome proliferator activated receptor gamma coactivator 1 alpha':ab,ti OR 'peroxisome proliferator-activated receptor gamma coactivator 1-alpha':ab,ti OR 'pgc 1 alpha':ab,ti OR 'pgc 1alpha':ab,ti OR 'ppar gamma coactivator 1 alpha':ab,ti OR 'ppar gamma coactivator 1alpha':ab,ti OR 'ppargc1a protein':ab,ti OR 'protein ppargc1a':ab,ti OR 'peroxisome proliferator activated receptor gamma coactivator 1alpha':ab,ti) AND ('sport'/exp OR 'competitive gymnastics':ab,ti OR 'competitive sport':ab,ti OR 'sports':ab,ti OR 'sport':ab,ti OR 'athlete'/exp OR 'athletes':ab,ti OR 'sportman':ab,ti OR 'sportmen':ab,ti OR 'sports player':ab,ti OR 'sports players':ab,ti OR 'sportsman':ab,ti OR 'sportsmen':ab,ti OR 'sportspeople':ab,ti OR 'sportsperson':ab,ti OR 'sportspersons':ab,ti OR 'sportsplayers':ab,ti OR 'sportswoman':ab,ti OR 'sportswomen':ab,ti OR 'sportwomen':ab,ti OR 'athlete':ab,ti) |
| Cochrane Library | #1 MeSH descriptor: [Peroxisome Proliferator-Activated Receptor Gamma Coactivator 1-alpha] explode all trees  #2 (PGC 1 alpha Protein):ti,ab,kw OR ( Peroxisome Proliferator Activated Receptor Gamma Coactivator 1 alpha):ti,ab,kw OR ( PPARGC 1 alpha Protein):ti,ab,kw OR ( PGC 1 alpha Protein):ti,ab,kw OR ( PPARGC1a Protein):ti,ab,kw OR ( PPARGC 1 alpha Protein):ti,ab,kw  #3 #1 OR #2  #4 MeSH descriptor: [Sports] explode all trees  #5 (Sport):ti,ab,kw OR (Athletic):ti,ab,kw OR (Athletics):ti,ab,kw  #6 MeSH descriptor: [Athletes] explode all trees  #7 (College Athletes):ti,ab,kw OR (Athlete, College):ti,ab,kw OR (Athletes, College):ti,ab,kw OR (College Athlete):ti,ab,kw OR (Athlete):ti,ab,kw OR (Athlete, Elite):ti,ab,kw OR (Elite Athletes):ti,ab,kw OR (Athletes, Elite):ti,ab,kw OR (Elite Athlete):ti,ab,kw OR (Professional Athletes):ti,ab,kw OR (Athlete, Professional):ti,ab,kw OR (Athletes, Professional):ti,ab,kw OR (Professional Athlete):ti,ab,kw  #8 #4 OR #5 OR #6 OR #7  #9 #3 AND #8 |

**Supplementary Table 2.** Distributions of the *PPARGC1A* Gly482Ser polymorphism genotypes in endurance athletes across included studies.

| Study | Group | Number | Genotype | | | Genotyping |
| --- | --- | --- | --- | --- | --- | --- |
|  |  |  | Gly/Gly | Gly/Ser | Ser/Ser |  |
| Bulğay et al. 2022 | Case  Control | 29  20 | 13  6 | 14  9 | 2  5 | WES |
| Elliott C.R. Hall et al. 2023 | Case  Control | 288  368 | 131  173 | 123  160 | 34  35 | qPCR |
| Eynon et al. 2011 | Case  Control | 74  240 | 37  79 | 37  117 | 0  44 | PCR-RFLP |
| Ginevicienė et al. 2011 | Case  Control | 77  250 | 40  129 | 33  104 | 4  17 | PCR-RFLP |
| Gineviciene et al. 2014 | Case  Control | 199  167 | 95  83 | 85  74 | 19  10 | PCR-RFLP |
| Grealy et al. 2015 | Case  Control | 195  113 | 74  51 | 84  45 | 37  17 | PCR-RFLP |
| Guilherme et al. 2018 | Case  Control | 316  893 | 153  428 | 140  385 | 23  80 | qPCR |
| He et al. 2015 | Case  Control | 235  504 | 73  156 | 115  244 | 47  104 | PCR-RFLP |
| Lucia et al. 2005 | Case  Control | 104  100 | 52  36 | 43  48 | 9  16 | FP-TDI |
| Maciejewska et al. 2012（Polish） | Case  Control | 92  684 | 52  280 | 36  314 | 4  90 | qPCR |
| Maciejewska et al. 2012（Russian） | Case  Control | 579  1132 | 294  489 | 247  505 | 38  138 | PCR-RFLP |
| Maruszak et al. 2012 | Case  Control | 213  413 | 95  185 | 100  195 | 18  33 | qPCR |
| Muniesa et al. 2010 | Case  Control | 141  123 | 65  47 | 52  63 | 24  13 | PCR-RFLP |
| Peplonska et al. 2017 | Case  Control | 225  451 | 102  199 | 105  213 | 18  39 | qPCR |
| Santiago et al. 2010 | Case  Control | 15  123 | 5  47 | 7  63 | 3  13 | PCR-RFLP |
| Varillas Delgado et al. 2020 | Case  Control | 123  122 | 77  65 | 46  49 | 0  8 | PCR-RFLP |
| Varillas Delgado et al. 2020 | Case  Control | 292  160 | 192  88 | 91  63 | 9  9 | PCR-SNPE |
| Yvert et al. 2016 | Case  Control | 154  649 | 39  191 | 77  324 | 38  134 | qPCR |

WES, Whole-exome sequencing; qPCR, real-time quantitative polymerase chain reaction; PCR-RFLP, polymerase chain reaction-restriction fragment length polymorphism; FP-TDI, Fluorescence polarization template-directed incorporation; PCR-SNPE, polymerase chain reaction-single nucleotide primer extension.

**Supplementary Table 3.** Distributions of the PPARGC1A Gly482Ser polymorphism genotypes in power athletes across included studies.

| Study | Group | Number | Genotype | | | Genotyping |
| --- | --- | --- | --- | --- | --- | --- |
|  |  |  | Gly/Gly | Gly/Ser | Ser/Ser |  |
| Bulğay et al. 2022 | Case  Control | 31  20 | 14  6 | 14  9 | 3  5 | WES |
| Eynon et al. 2011 | Case  Control | 81  240 | 35  79 | 36  117 | 10  44 | PCR-RFLP |
| Ginevicienė et al. 2011 | Case  Control | 51  250 | 29  129 | 21  104 | 1  17 | PCR-RFLP |
| Gineviciene et al. 2016（Russian） | Case  Control | 114  947 | 62  424 | 35  416 | 17  107 | PCR-RFLP |
| Gineviciene et al. 2016（Lithuanian） | Case  Control | 47  255 | 24  132 | 22  106 | 1  17 | PCR-RFLP |
| Guilherme and Lancha, 2020 | Case  Control | 83  818 | 47  387 | 32  357 | 4  74 | qPCR |
| Guilherme et al. 2018 | Case  Control | 314  893 | 173  428 | 116  385 | 25  80 | qPCR |
| Homma et al. 2022 (power athletes) | Case  Control | 177  416 | 51  153 | 94  184 | 32  79 | qPCR |
| Homma et al. 2022 (weightlifters) | Case  Control | 192  416 | 55  153 | 102  184 | 35  79 | qPCR |
| Maciejewska et al. 2012（Polish） | Case  Control | 210  684 | 118  280 | 79  314 | 13  90 | qPCR |
| Maciejewska et al. 2012（Russian） | Case  Contro | 724  1132 | 329  489 | 322  505 | 73  138 | PCR-RFLP |
| Maruszak et al. 2012 | Case  Control | 182  413 | 95  185 | 70  195 | 17  33 | qPCR |
| Peplonska et al. 2017 | Case  Control | 188  451 | 97  199 | 73  213 | 18  39 | qPCR |
| Valipour et al. 2021 | Case  Control | 50  100 | 17  40 | 27  48 | 6  12 | PCR-RFLP |

WES, Whole-exome sequencing; qPCR, real-time quantitative polymerase chain reaction; PCR-RFLP, polymerase chain reaction-restriction fragment length polymorphism.


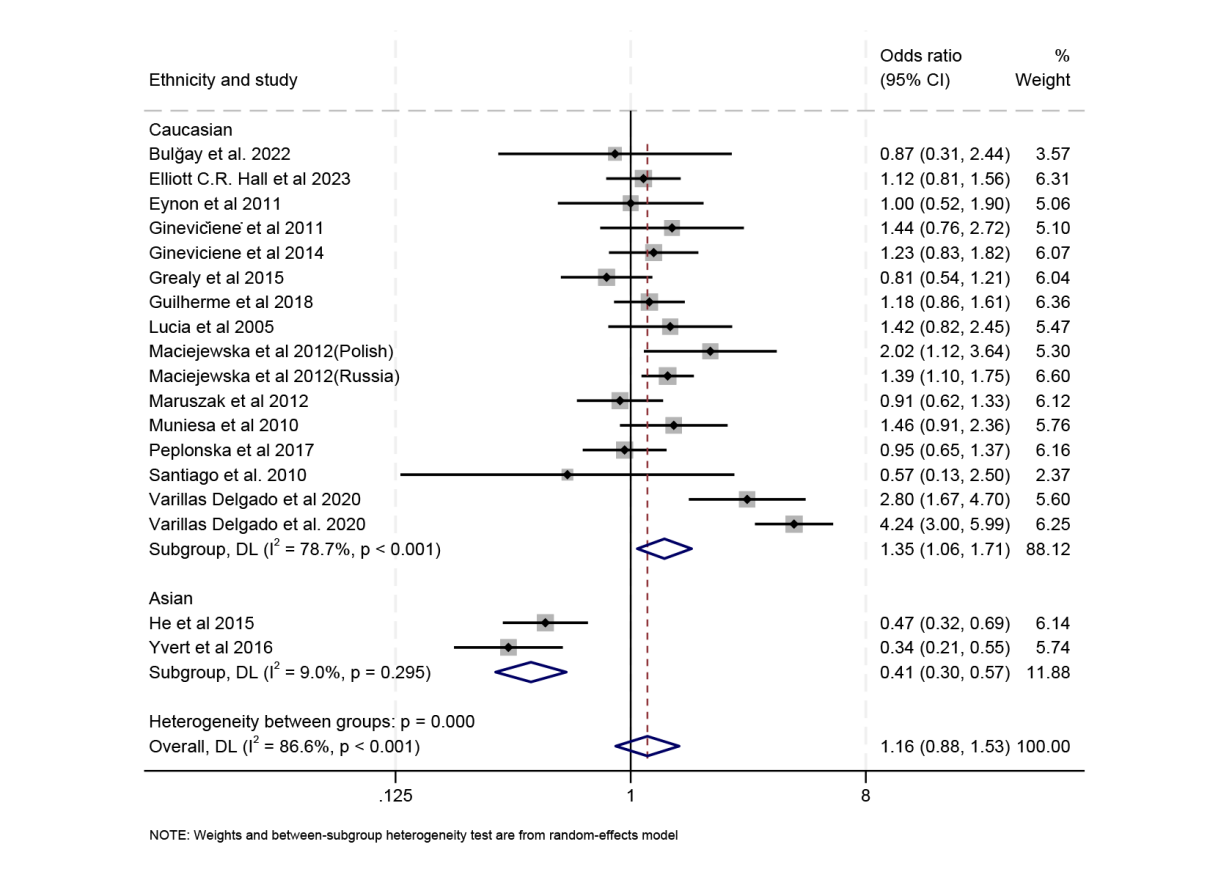


**Supplementary Figure 1.** Forest plot of the comparison between frequencies of Gly/Gly and Gly/Ser genotypes in endurance athletes.


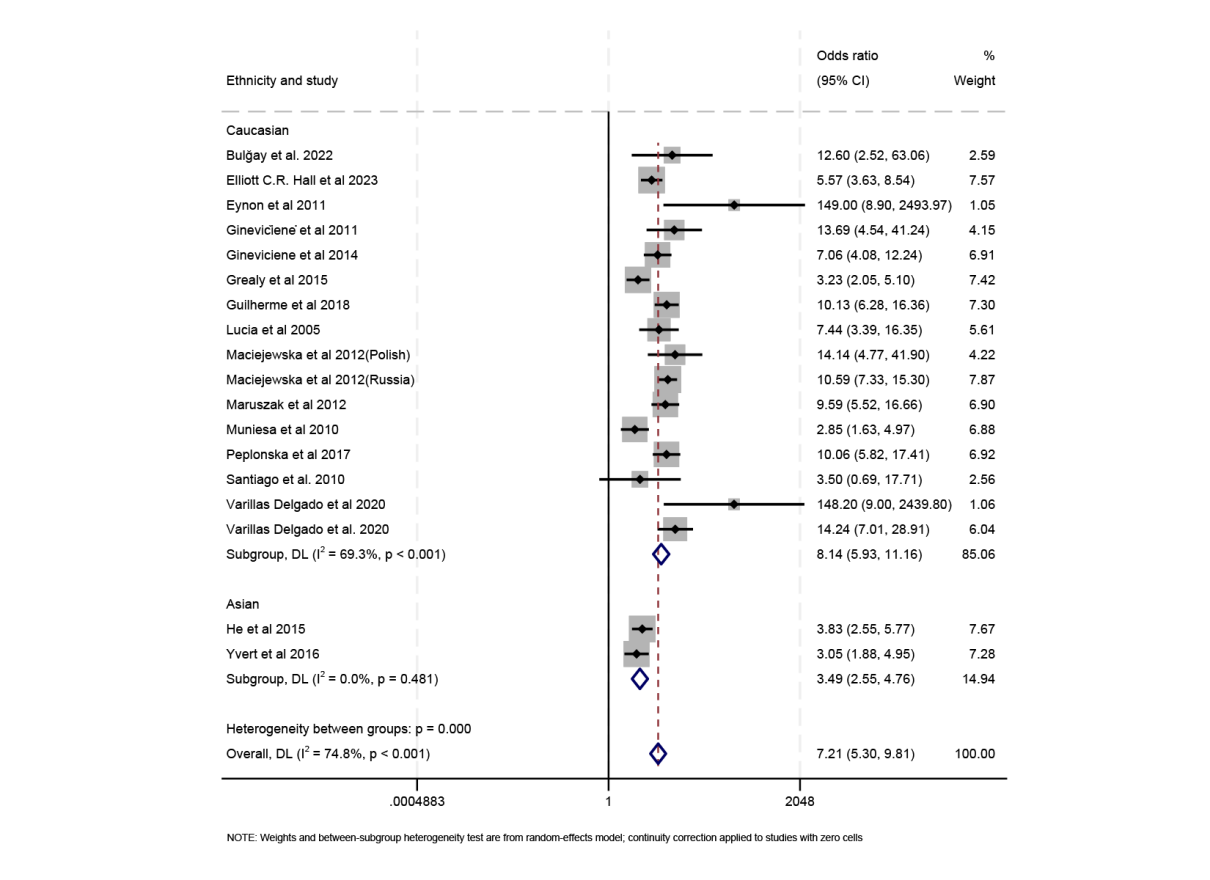


**Supplementary Figure 2.** Forest plot of the comparison between frequencies of Gly/Ser and Ser/Ser genotypes in endurance athletes.


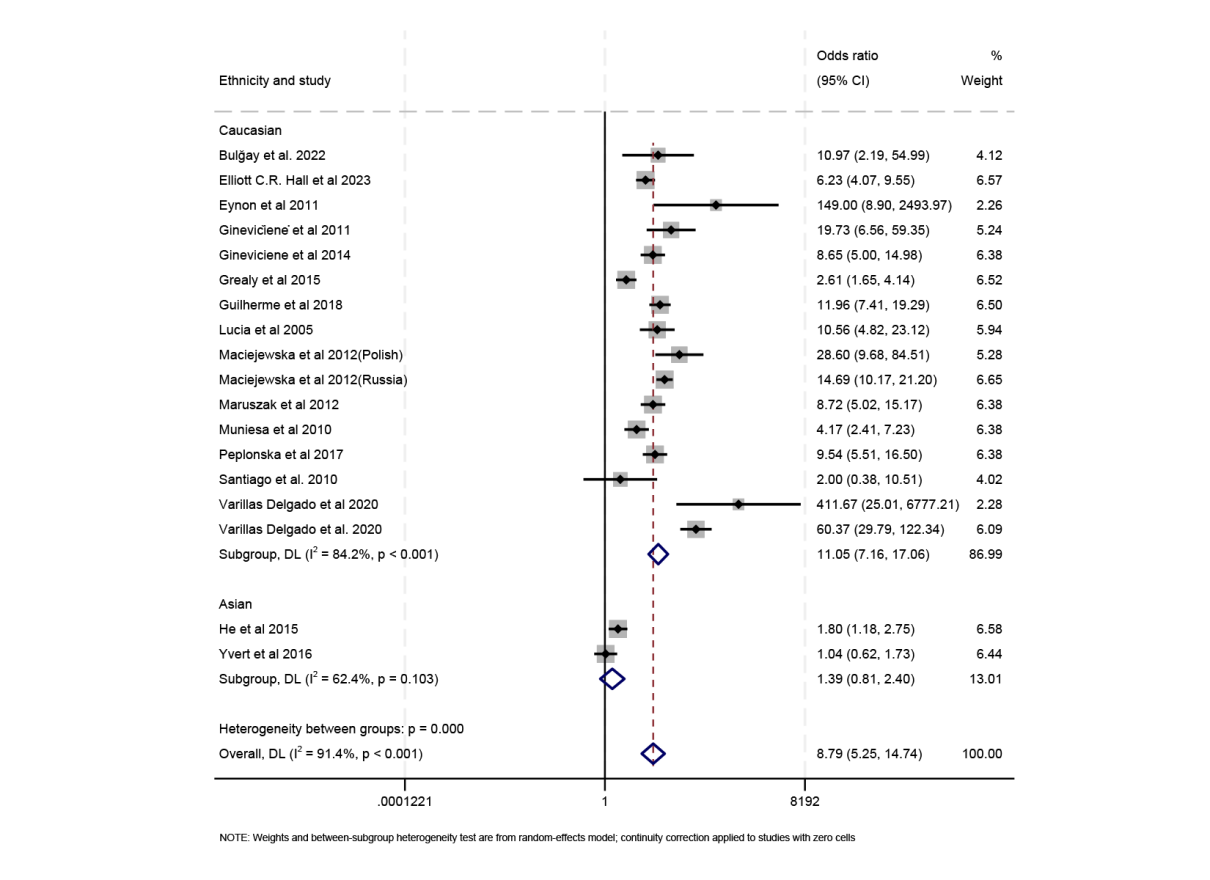


**Supplementary Figure 3.** Forest plot of the comparison between frequencies of Gly/Gly and Ser/Ser genotypes in endurance athletes.


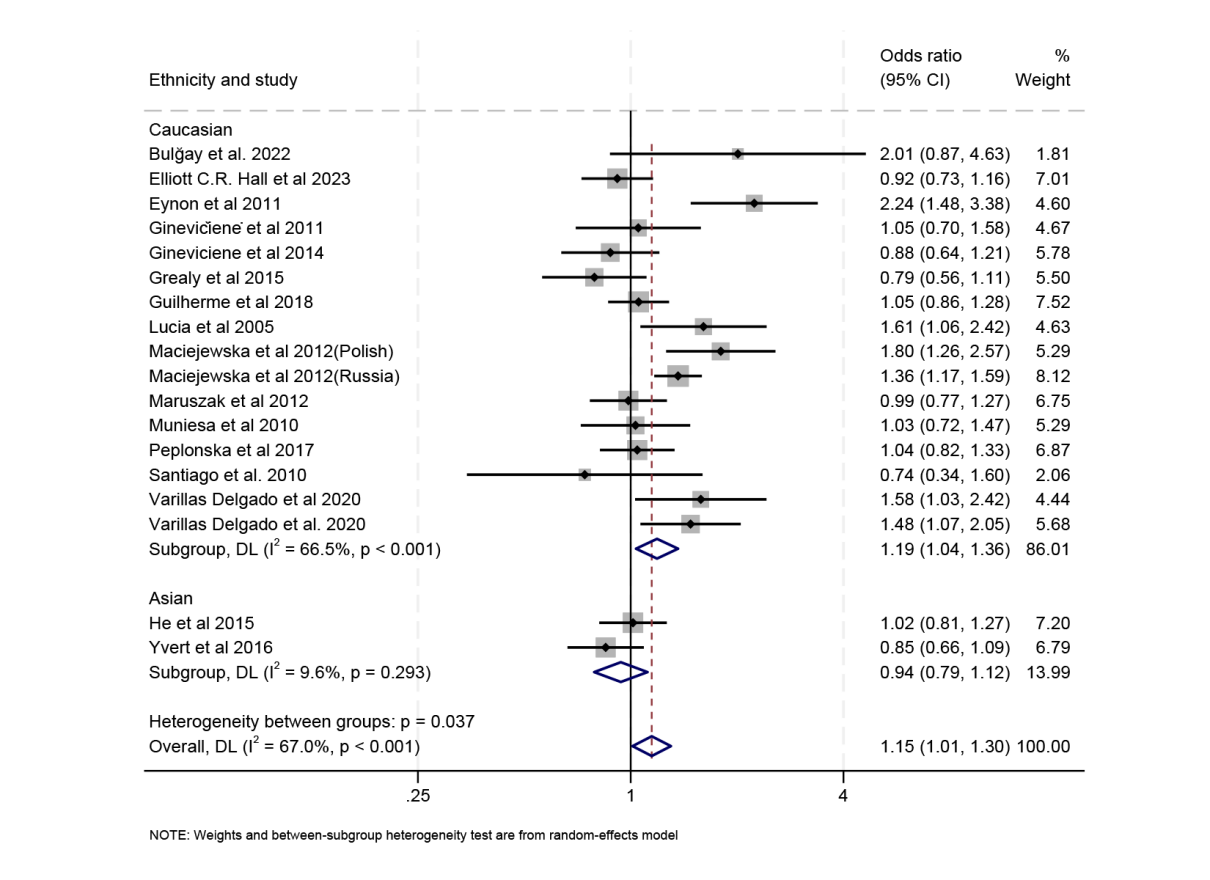


**Supplementary Figure 4.** Forest plot of the comparison between frequencies of the Gly allele and the Ser allele in endurance athletes versus controls.


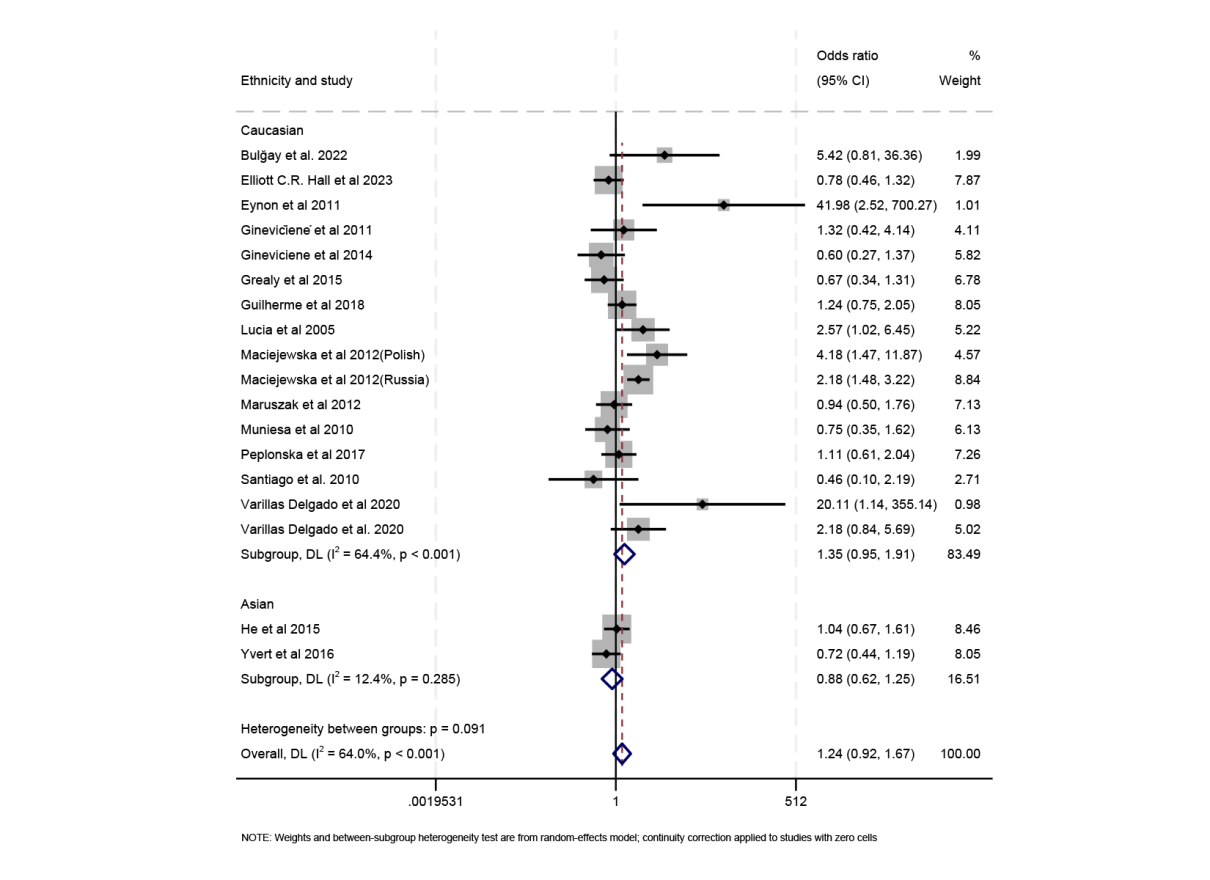


**Supplementary Figure 5.** Forest plot of the comparison between frequencies of Gly/Gly and Ser/Ser genotypes in endurance athletes versus controls


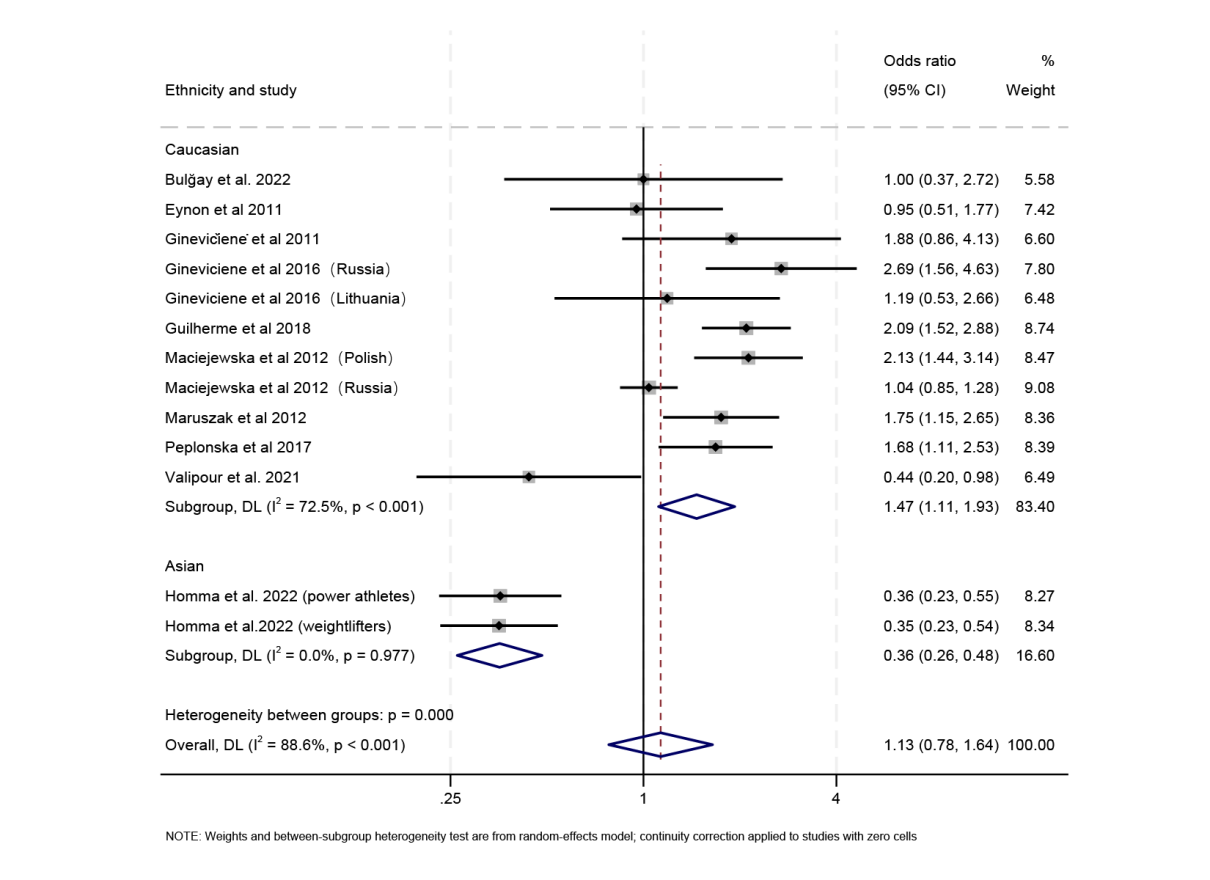


**Supplementary Figure 6.** Forest plot of the comparison between frequencies of Gly/Gly and Gly/Ser genotypes in power athletes.


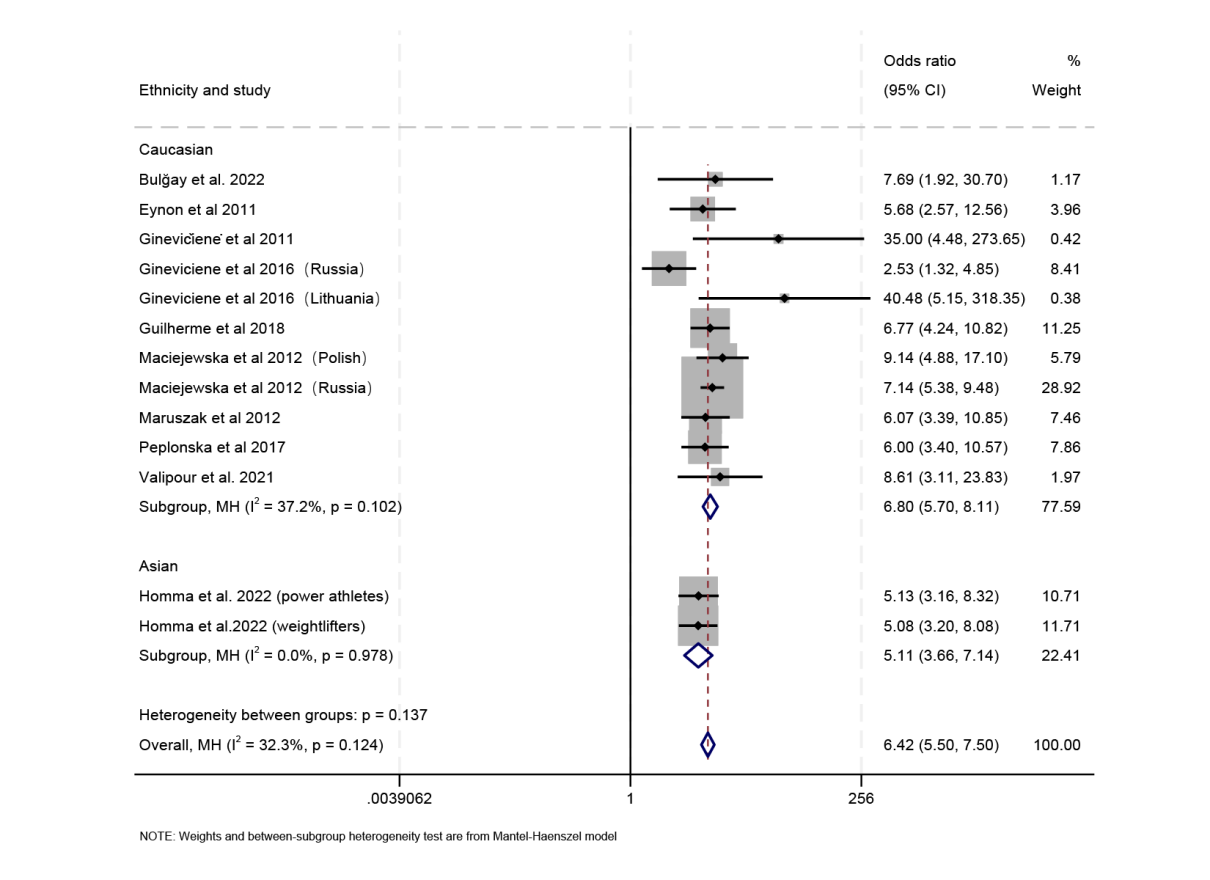


**Supplementary Figure 7.** Forest plot of the comparison between frequencies of Gly/Ser and Ser/Ser genotypes in power athletes.


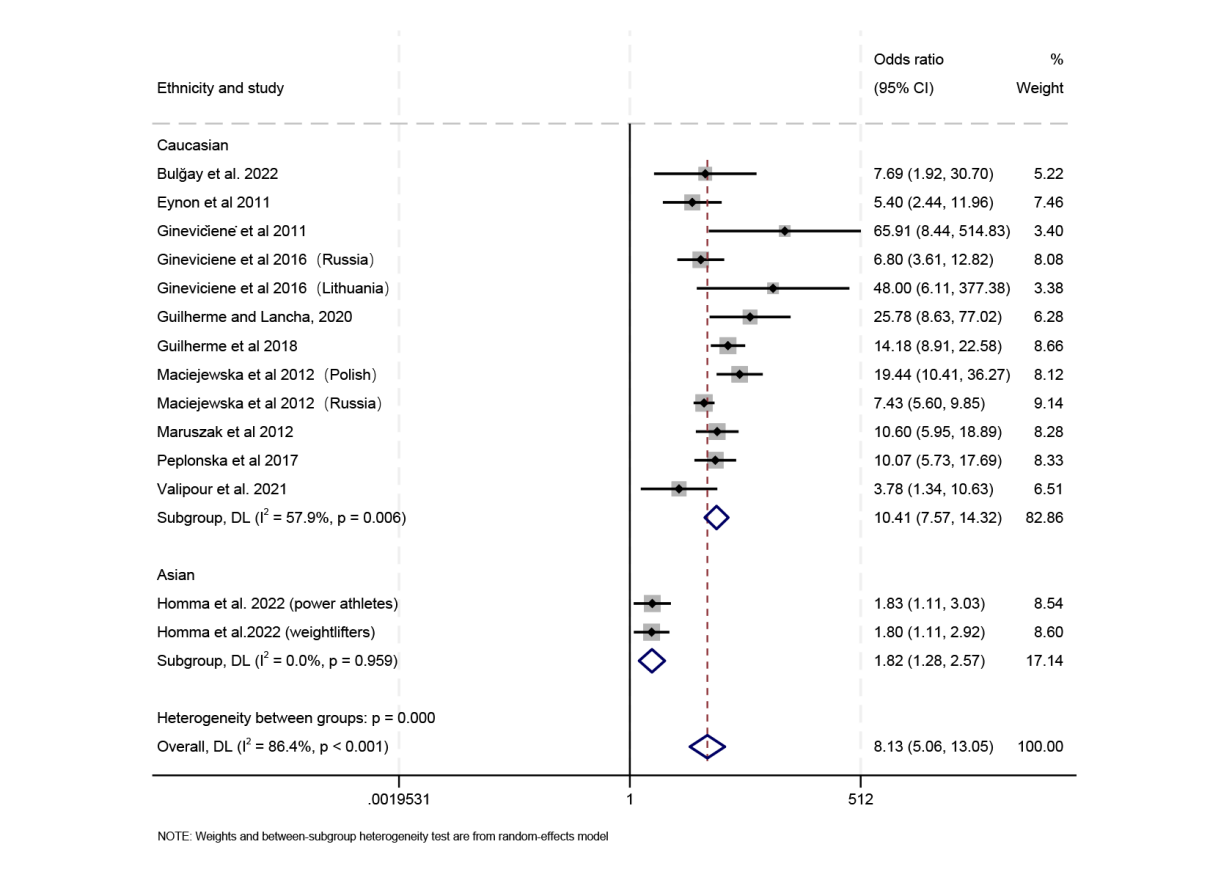


**Supplementary Figure 8.** Forest plot of the comparison between frequencies of Gly/Gly and Ser/Ser genotypes in power athletes.


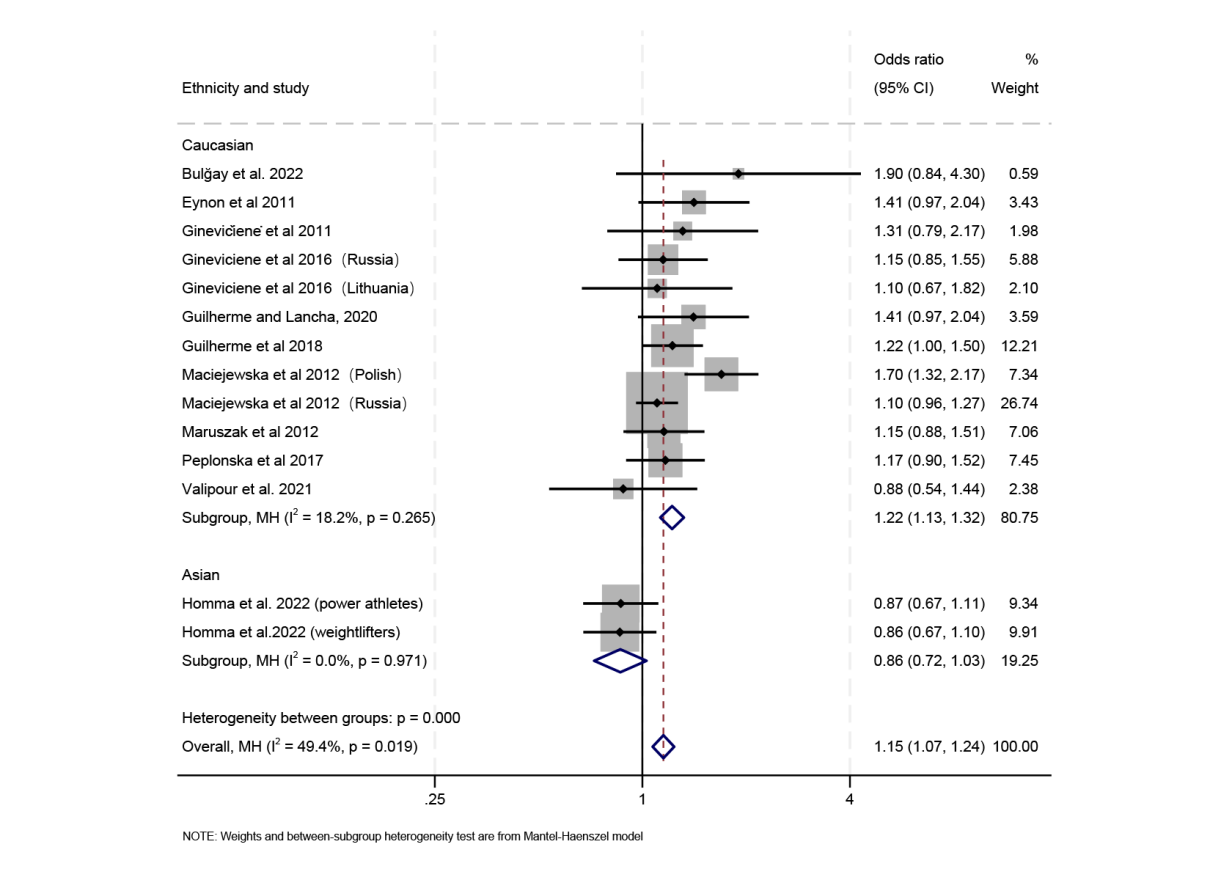


**Supplementary Figure 9.** Forest plot of the comparison between frequencies of the Gly allele and the Ser allele in power athletes versus controls.


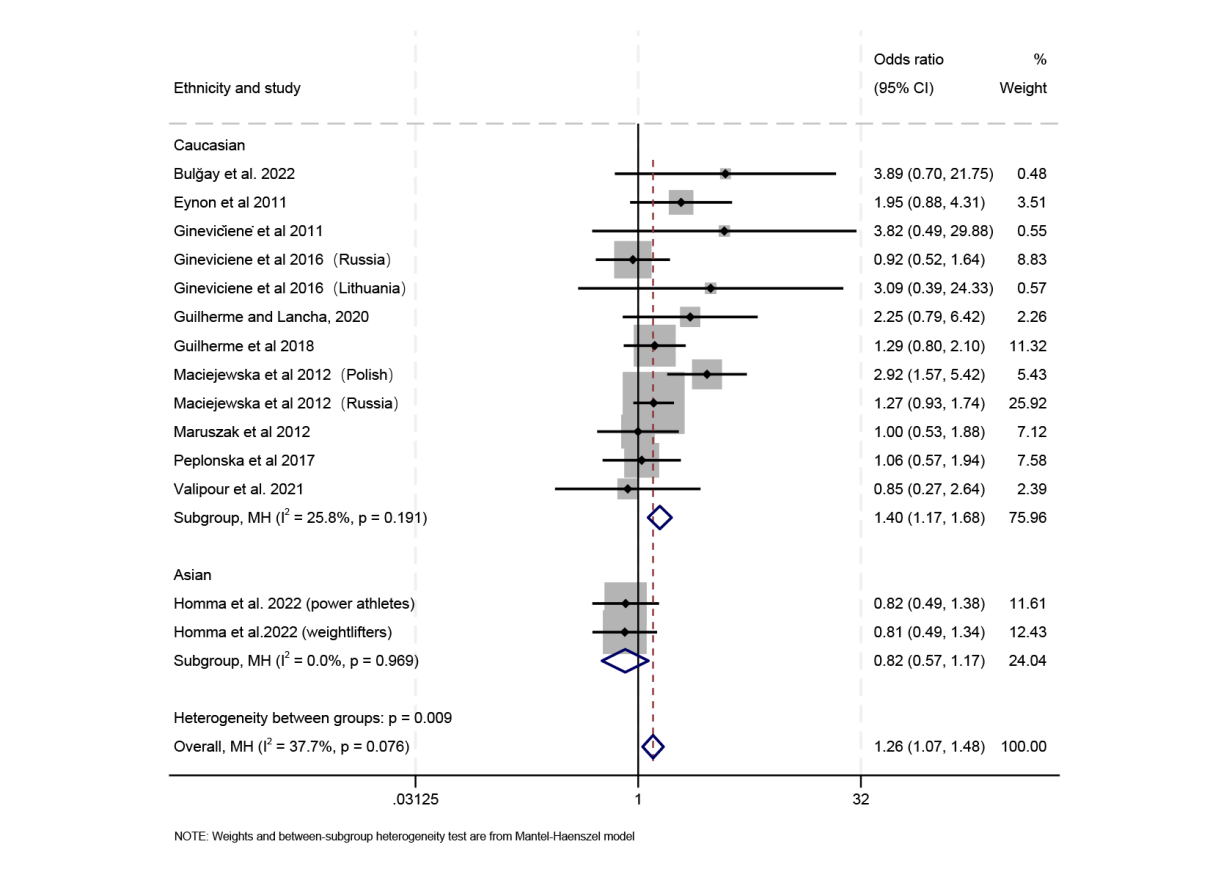


**Supplementary Figure 10.** Forest plot of the comparison between frequencies of Gly/Gly and Ser/Ser genotypes in power athletes versus controls.


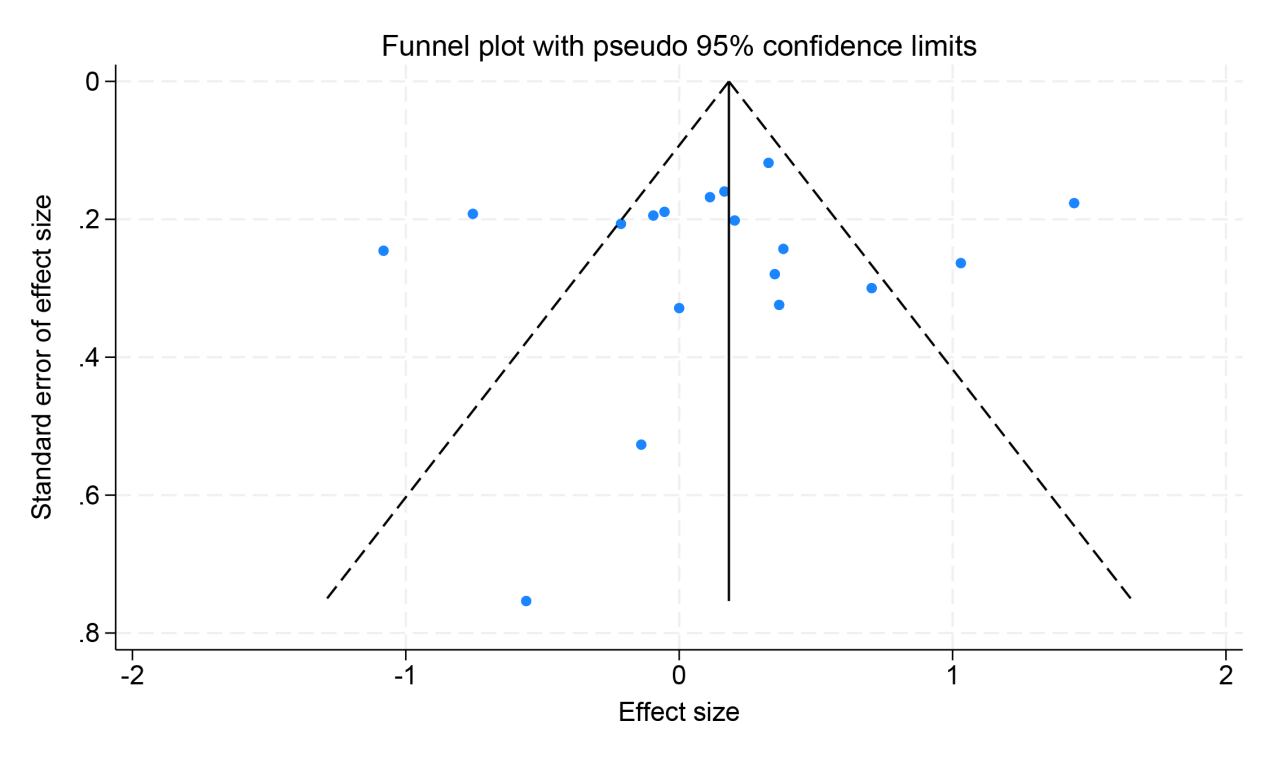


**Supplementary Figure 11.** Funnel plot of the comparison between frequencies of Gly/Gly and Gly/Ser genotypes in endurance athletes.


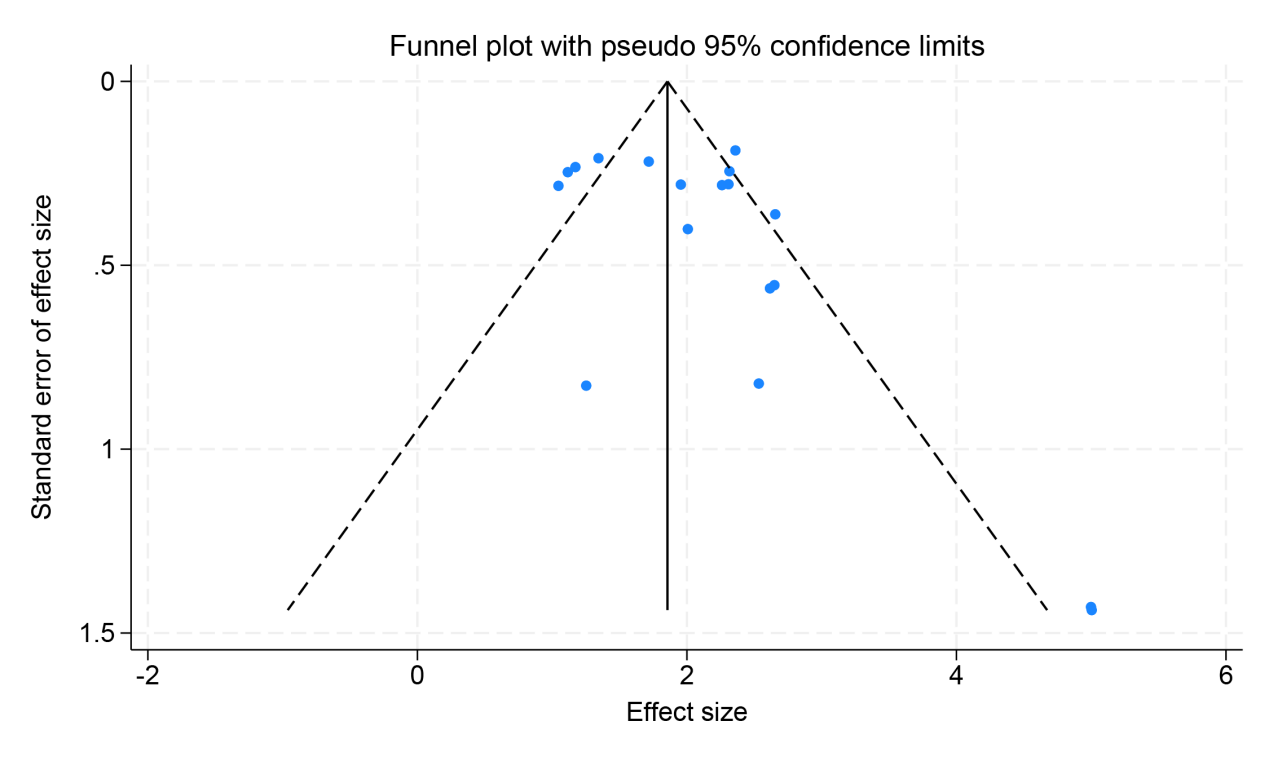


**Supplementary Figure 12.** Funnel plot of the comparison between frequencies of Gly/Ser and Ser/Ser genotypes in endurance athletes.


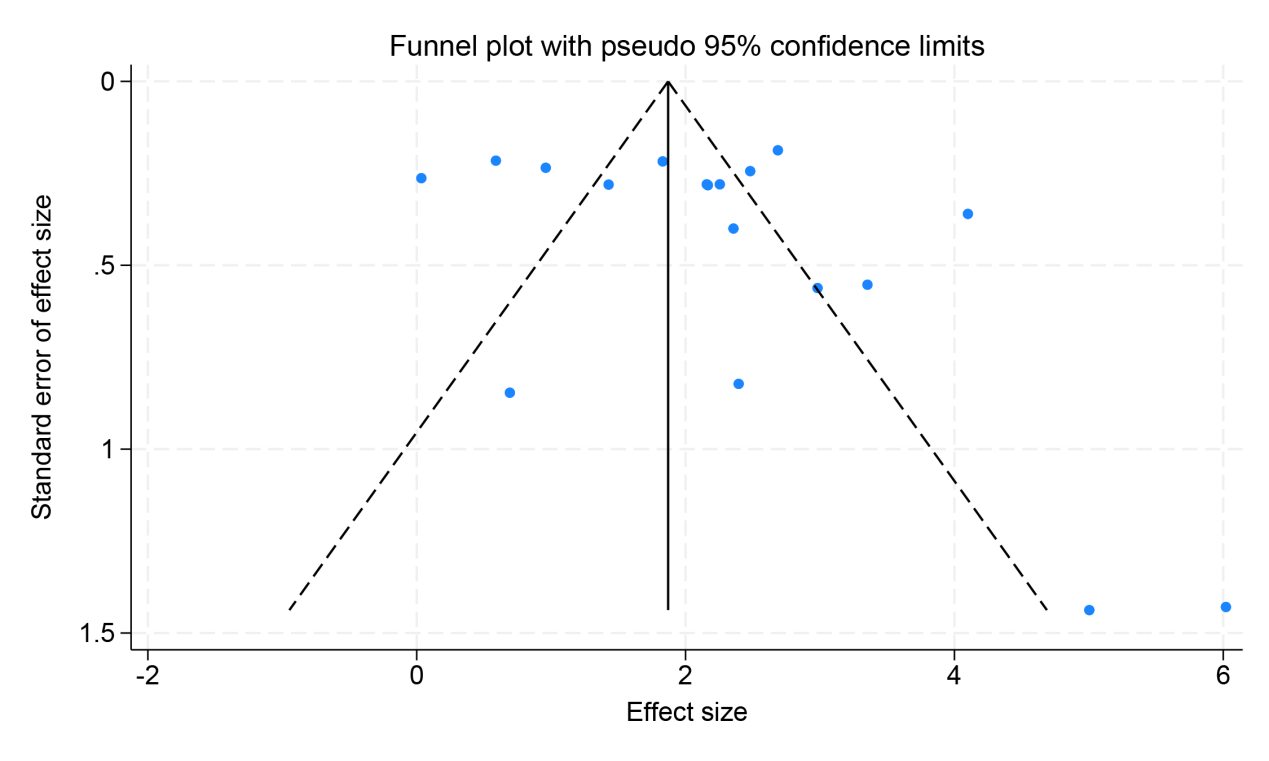


**Supplementary Figure 13.** Funnel plot of the comparison between frequencies of Gly/Gly and Ser/Ser genotypes in endurance athletes.


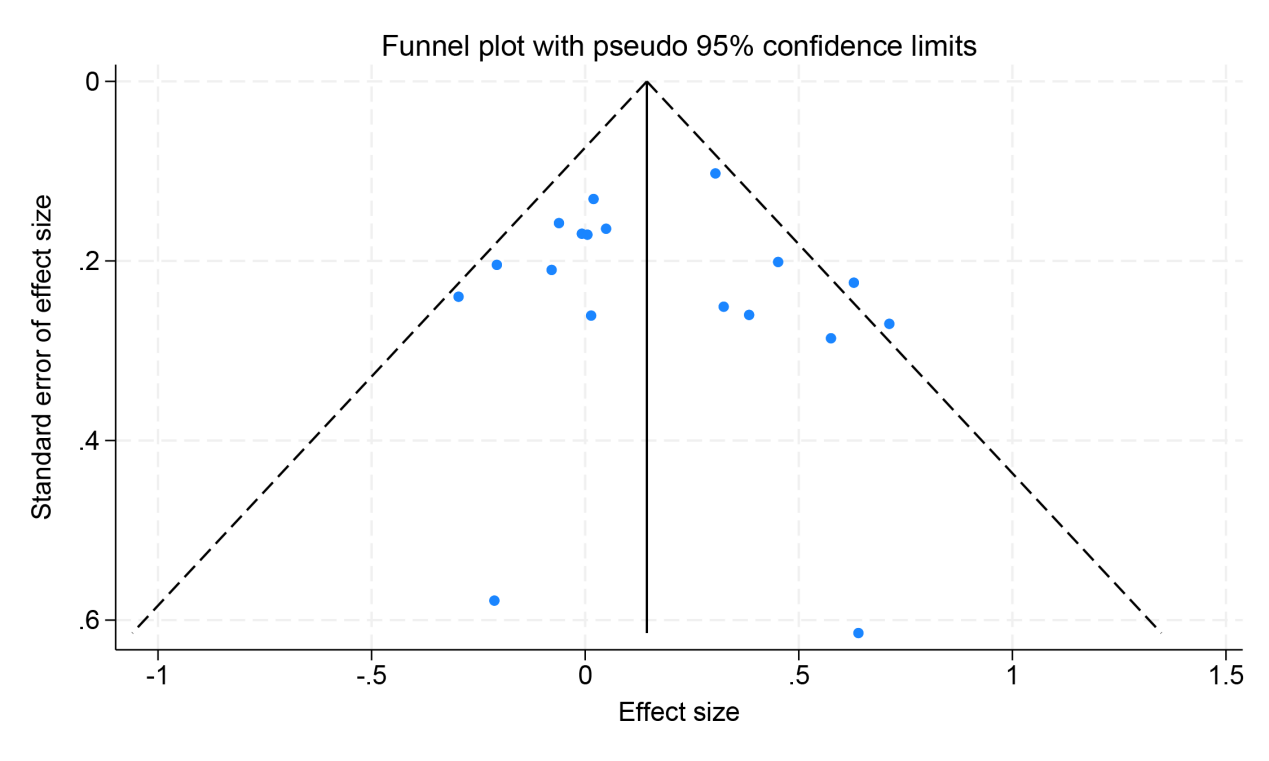


**Supplementary Figure 14.** Funnel plot of the comparison between frequencies of Gly/Gly and Gly/Ser+Ser/Ser genotypes in endurance athletes versus controls


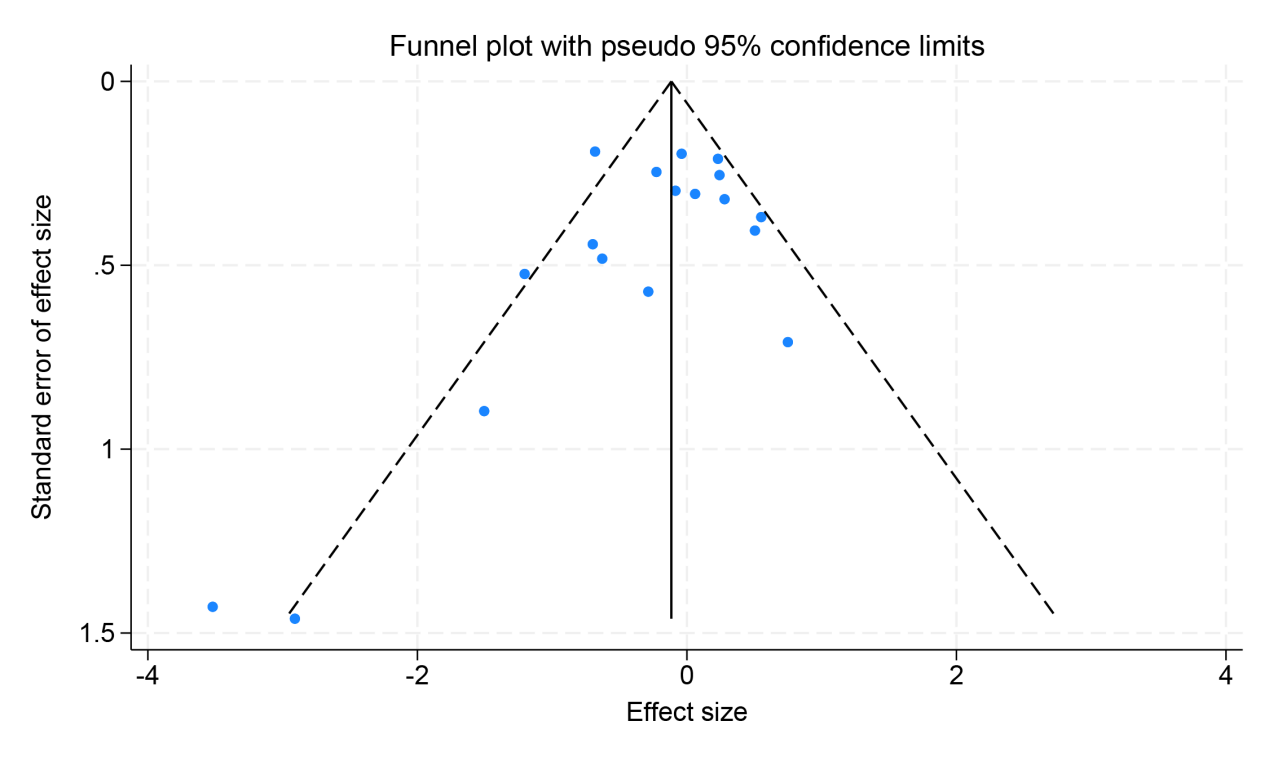


**Supplementary Figure 15.** Funnel plot of the comparison between frequencies of Ser/Ser and Gly/Gly+Gly/Ser genotypes in endurance athletes versus controls.


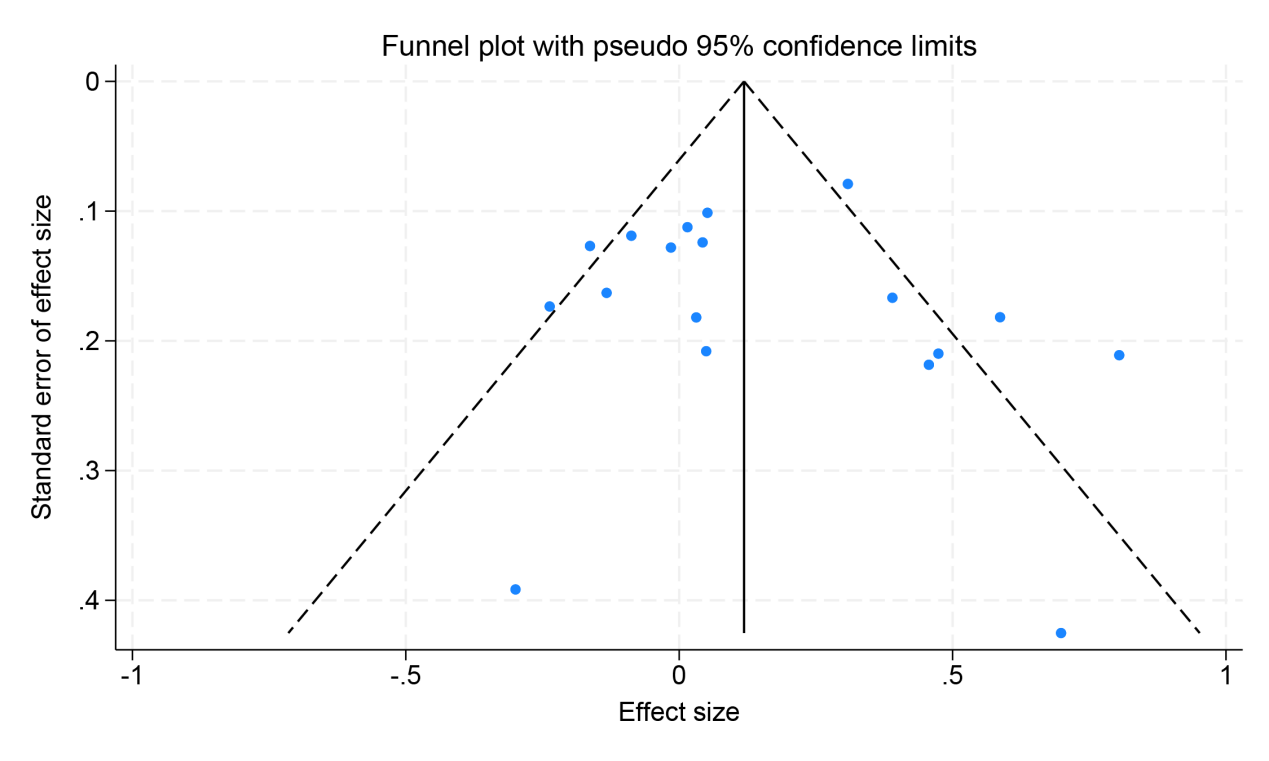


**Supplementary Figure 16.** Funnel plot of the comparison between frequencies of the Gly allele and the Ser allele in endurance athletes versus controls.


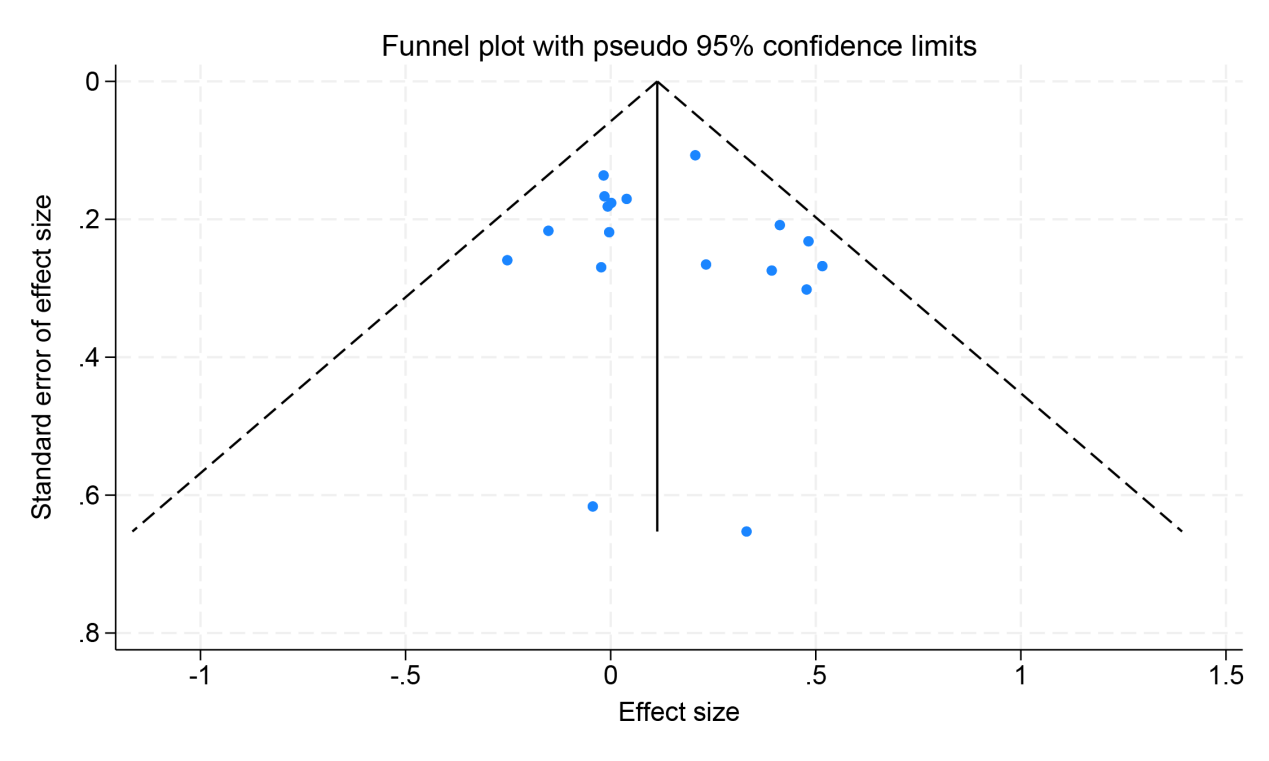


**Supplementary Figure 17.** Funnel plot of the comparison between frequencies of Gly/Gly and Gly/Ser genotypes in endurance athletes.


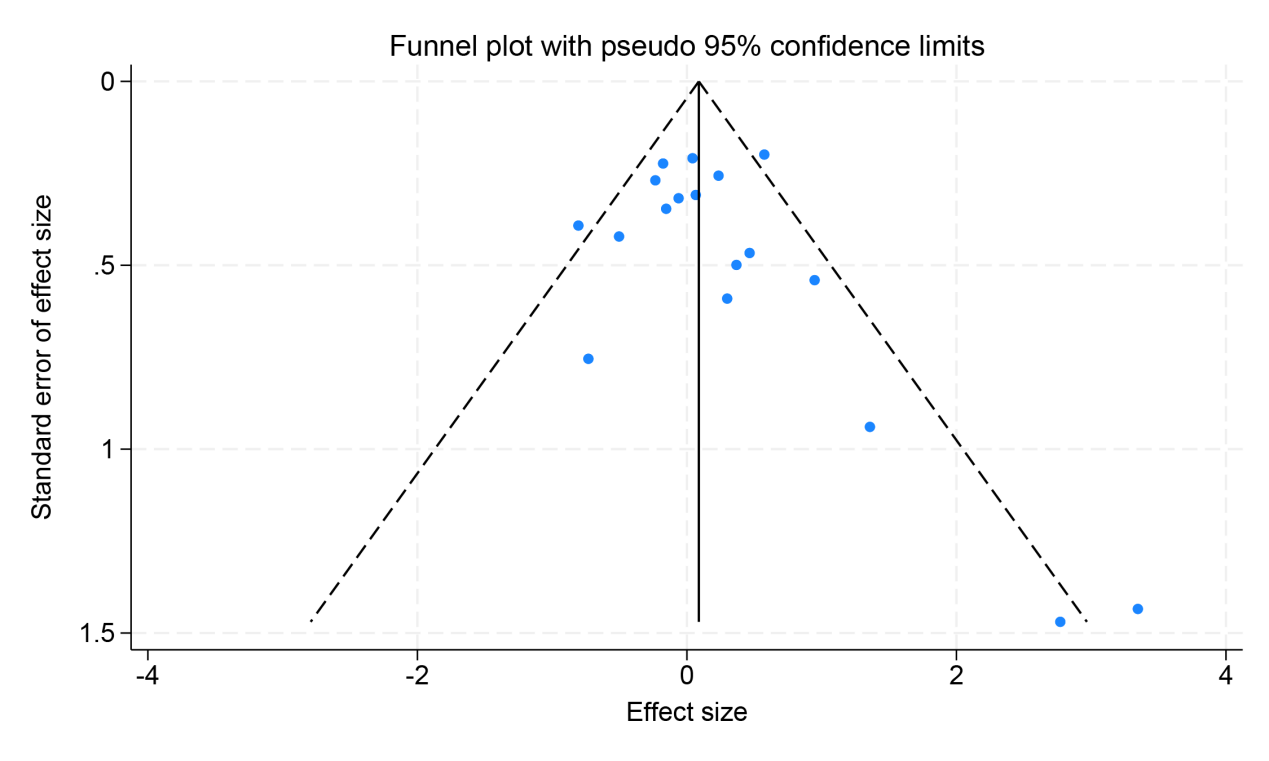


**Supplementary Figure 18.** Funnel plot of the comparison between frequencies of Gly/Ser and Ser/Ser genotypes in endurance athletes.


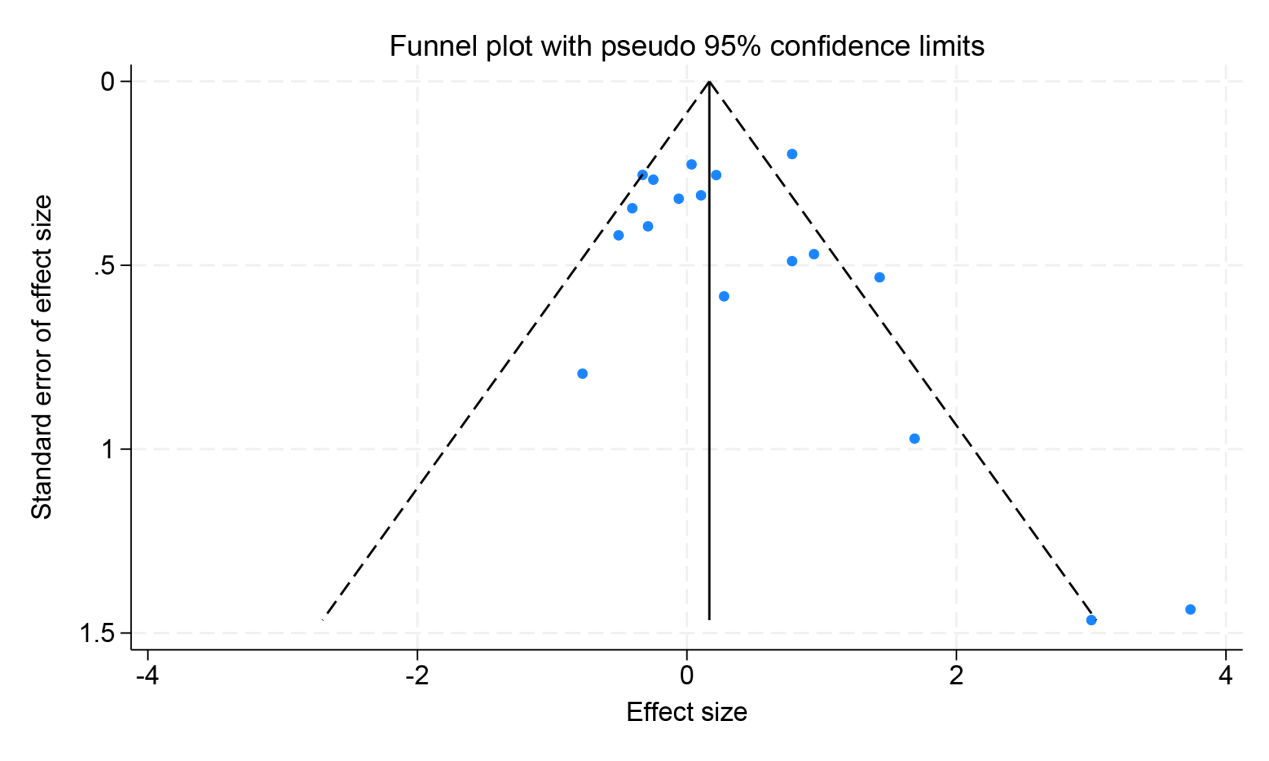


**Supplementary Figure 19.** Funnel plot of the comparison between frequencies of Gly/Gly and Ser/Ser genotypes in endurance athletes.


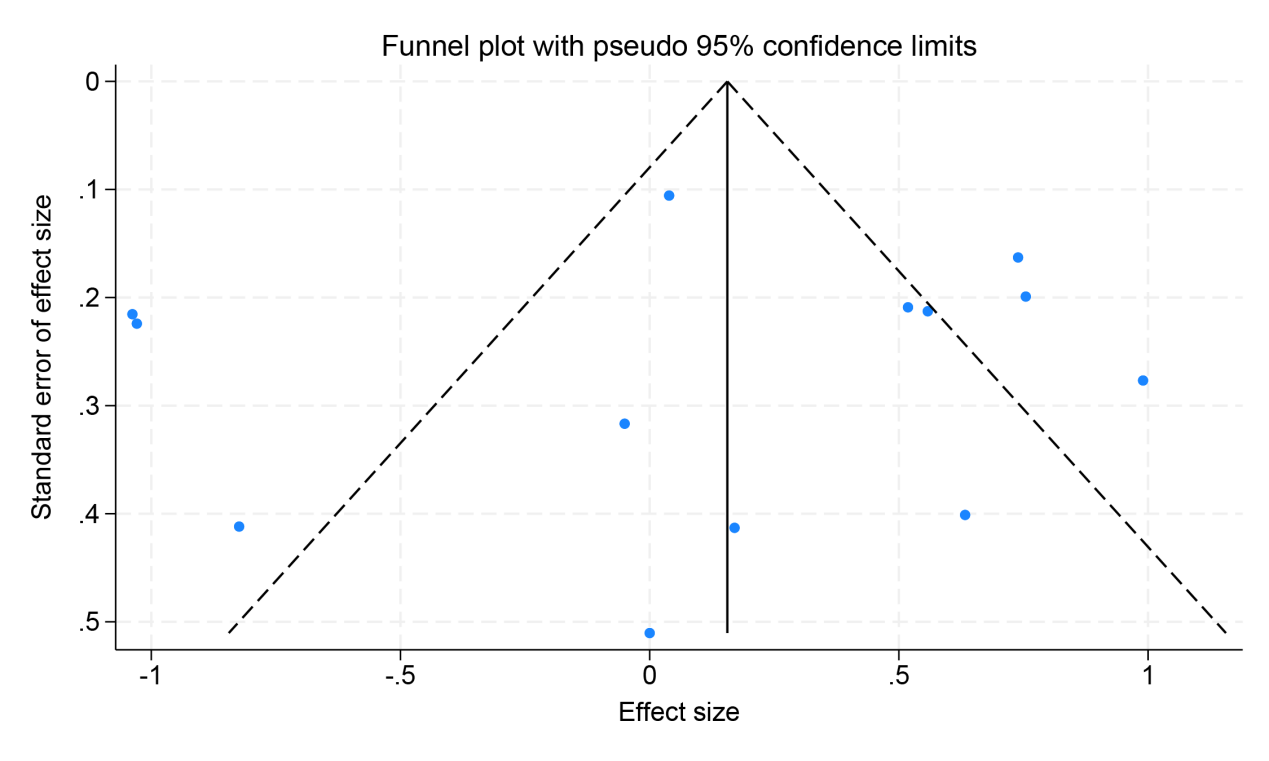


**Supplementary Figure 20.** Funnel plot of the comparison between frequencies of Gly/Gly and Gly/Ser genotypes in power athletes.


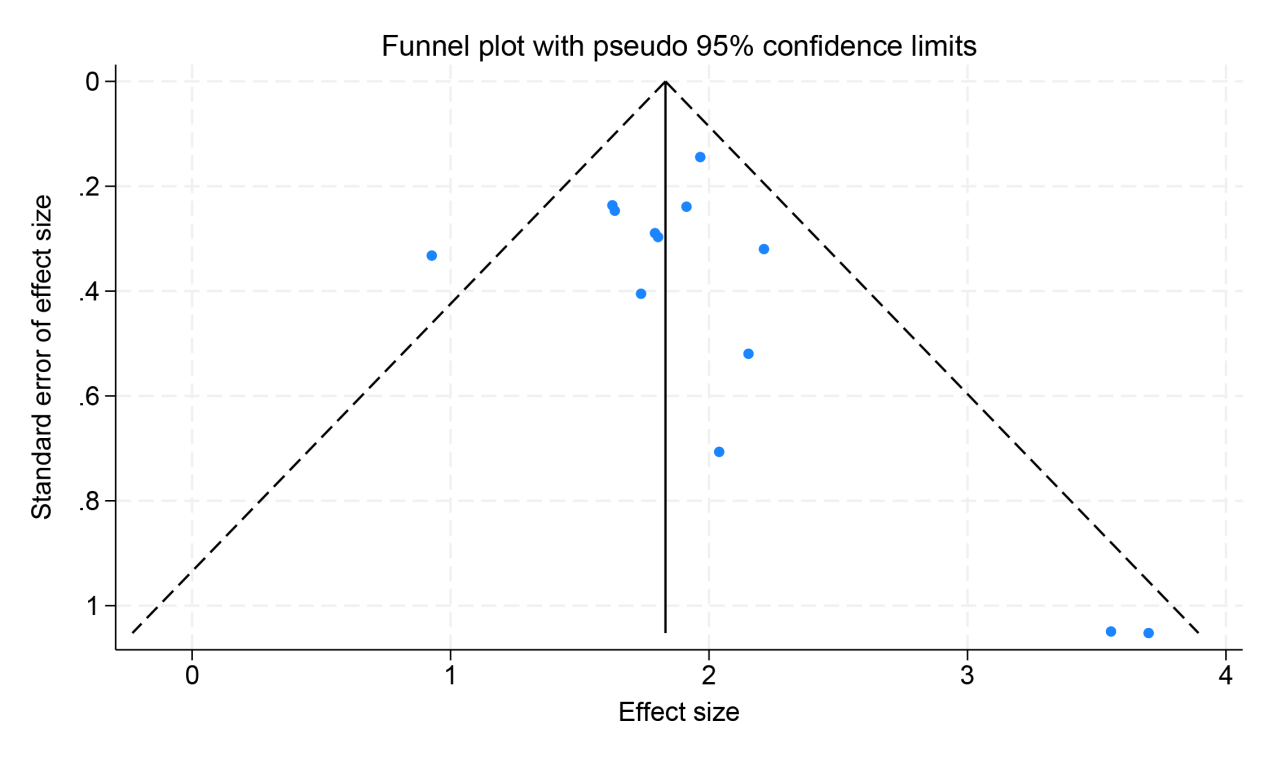


**Supplementary Figure 21.** Funnel plot of the comparison between frequencies of Gly/Ser and Ser/Ser genotypes in power athletes.


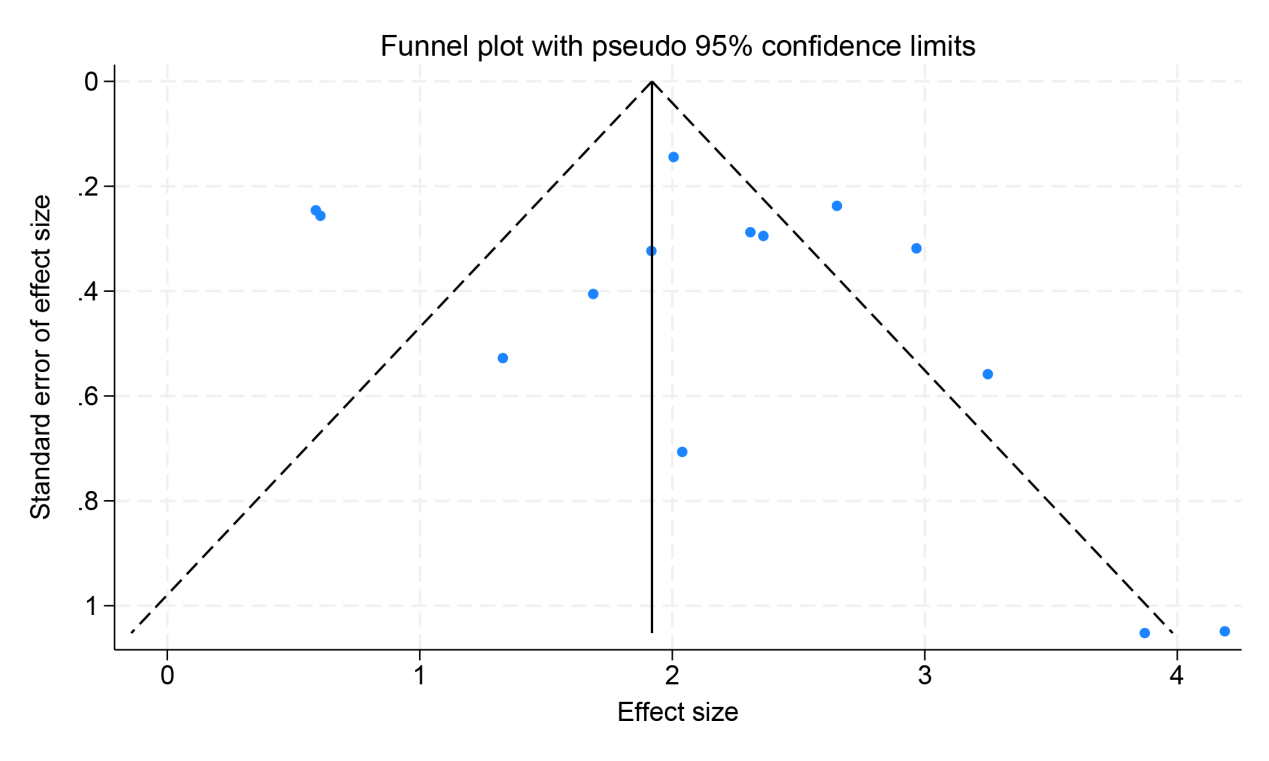


**Supplementary Figure 22.** Funnel plot of the comparison between frequencies of Gly/Gly and Ser/Ser genotypes in power athletes.


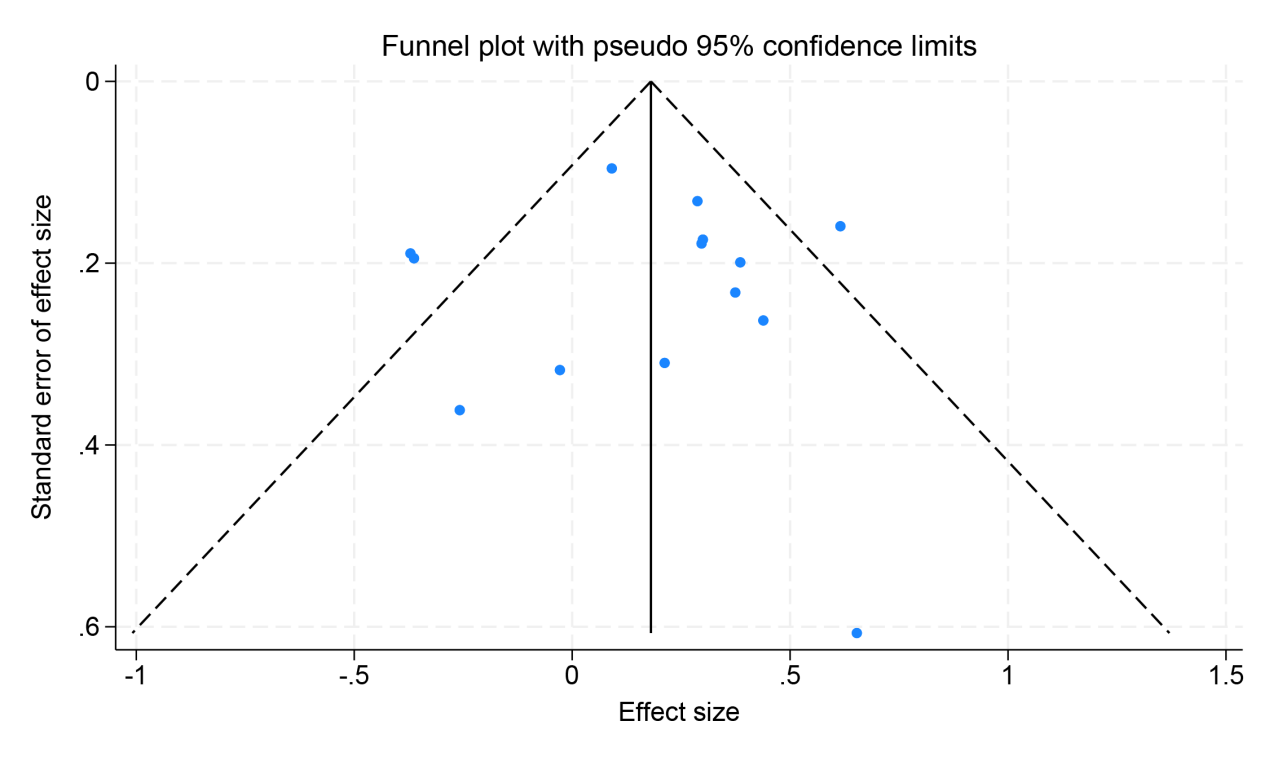


**Supplementary Figure 23.** Funnel plot of the comparison between frequencies of Gly/Gly and Gly/Ser + Ser/Ser genotypes in power athletes versus controls.


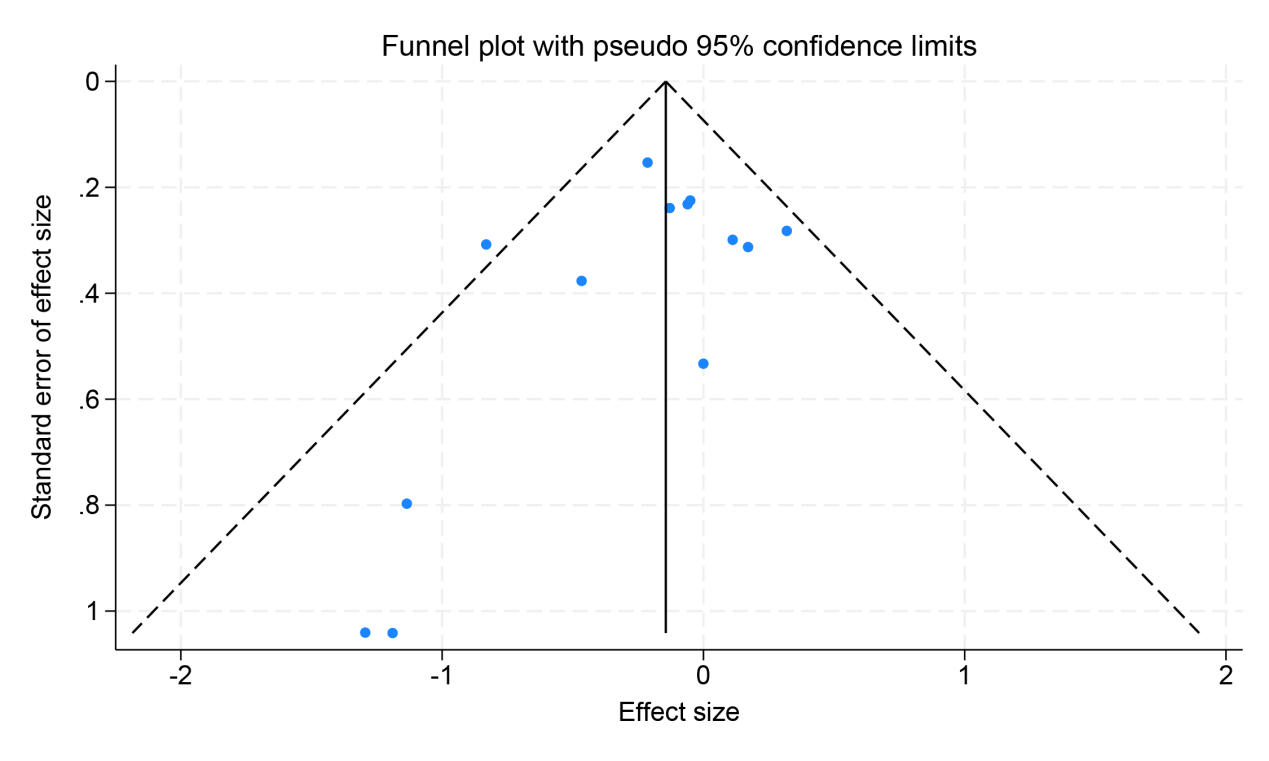


**Supplementary Figure 24.** Funnel plot of the comparison between frequencies of Ser/Ser and Gly/Gly + Gly/Ser genotypes in power athletes versus controls.


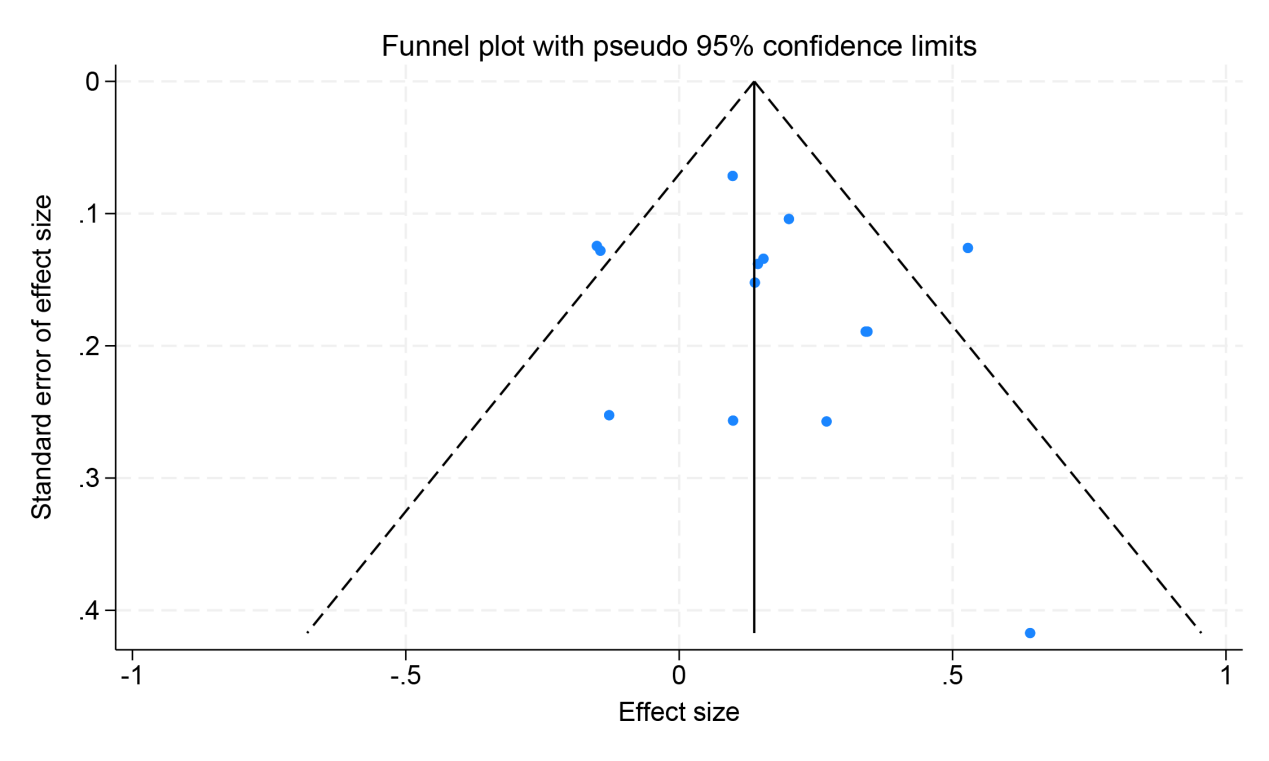


**Supplementary Figure 25.** Forest plot of the comparison between frequencies of the Gly allele and the Ser allele in power athletes versus controls.


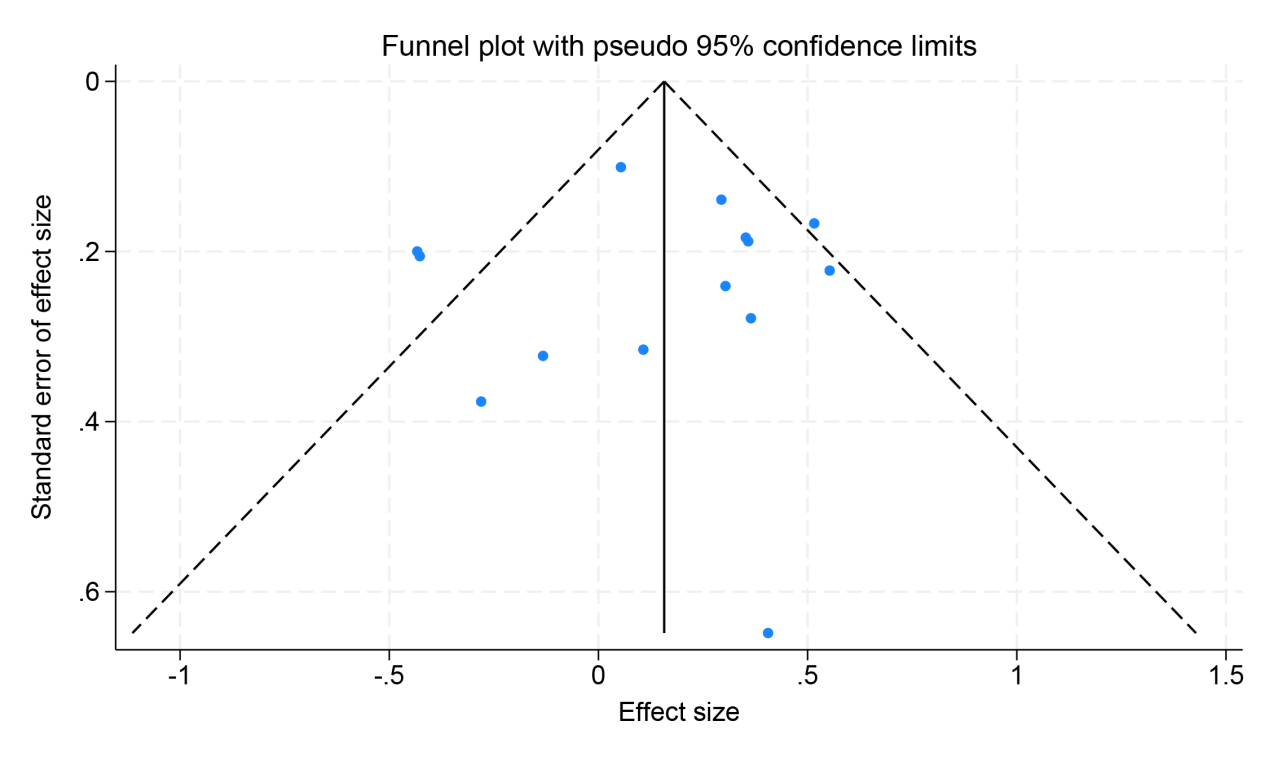


**Supplementary Figure 26.** Funnel plot of the comparison between frequencies of Gly/Gly and Gly/Ser genotypes in power athletes versus controls.


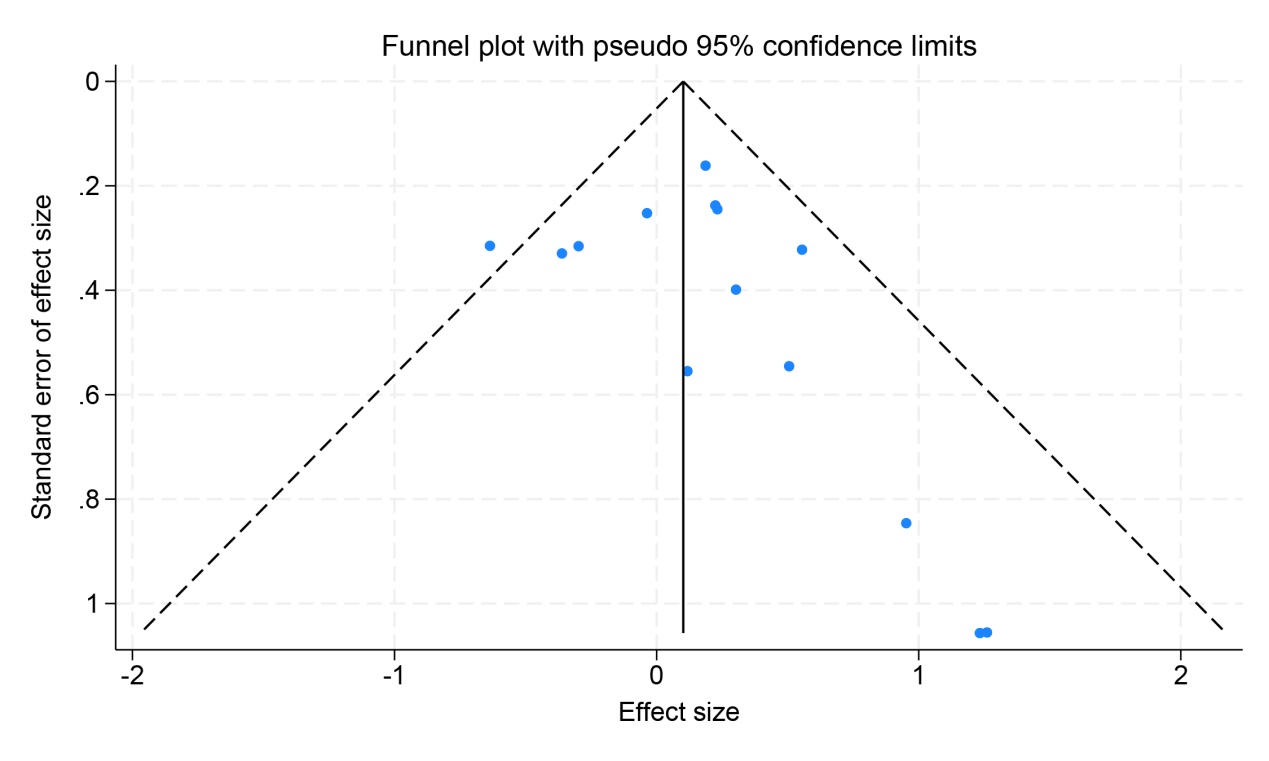


**Supplementary Figure 27.** Funnel plot of the comparison between frequencies of Gly/Ser and Ser/Ser genotypes in power athletes versus controls.


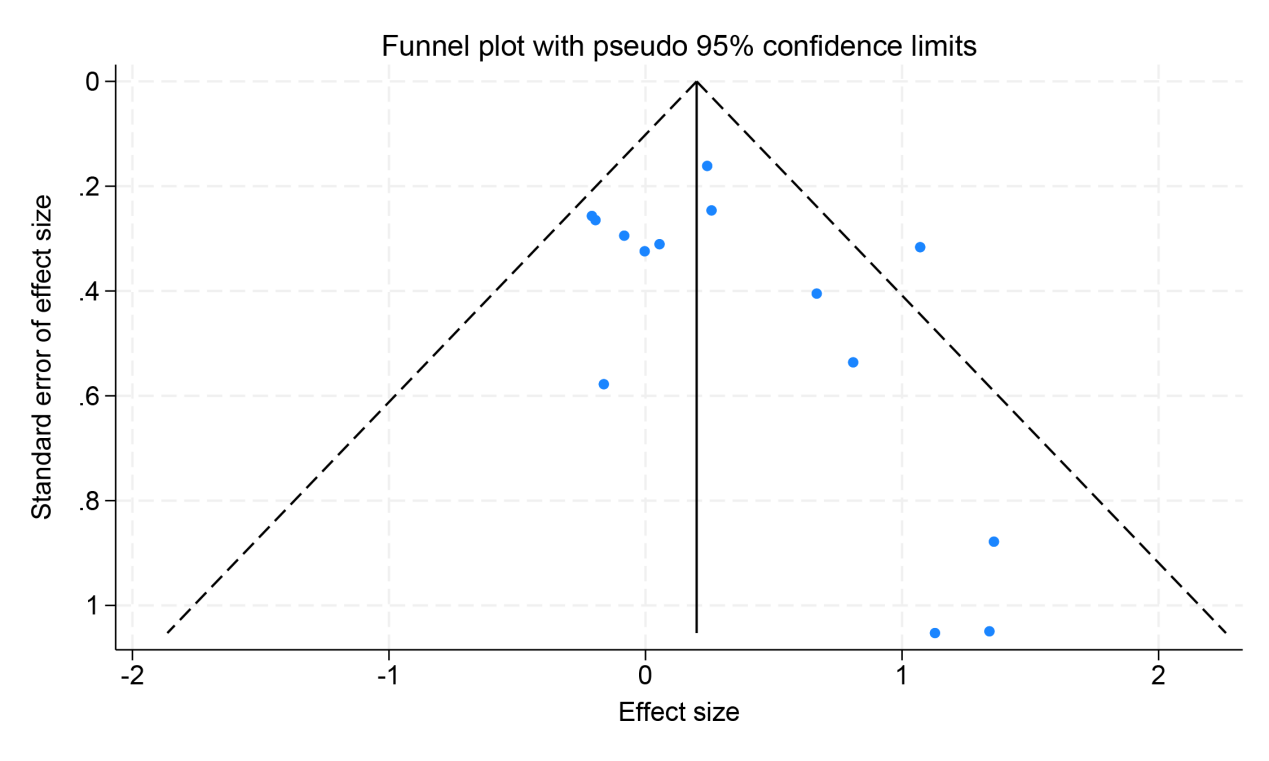


**Supplementary Figure 28.** Funnel plot of the comparison between frequencies of Gly/Gly and Ser/Ser genotypes in power athletes versus controls.
